# Investigating key factors underlying neurodegeneration linked to alpha‐synuclein spread

**DOI:** 10.1111/nan.12829

**Published:** 2022-07-04

**Authors:** Cindy C. C. Pang, Maja H. Sørensen, Krit Lee, Kelvin C. Luk, John Q. Trojanowski, Virginia M. Y. Lee, Wendy Noble, Raymond C. C. Chang

**Affiliations:** ^1^ Laboratory of Neurodegenerative Diseases, School of Biomedical Sciences, LKS Faculty of Medicine The University of Hong Kong Hong Kong SAR China; ^2^ Institute of Psychiatry, Psychology and Neuroscience, Department of Basic and Clinical Neuroscience King's College London London UK; ^3^ Center for Neurodegenerative Disease Research, Department of Pathology and Laboratory Medicine, Alzheimer's Disease Core Center, Institute on Aging University of Pennsylvania Perelman School of Medicine Philadelphia Pennsylvania USA; ^4^ State Key Laboratory of Brain and Cognitive Sciences The University of Hong Kong Pokfulam Hong Kong SAR China

**Keywords:** 6‐OHDA, alpha‐synuclein, Parkinson's disease, oxidative stress, Tau

## Abstract

**Aims:**

It has long been considered that accumulation of pathological alpha‐synuclein (aSyn) leads to synaptic/neuronal loss which then results in behavioural and cognitive dysfunction. To investigate this claim, we investigated effects downstream of aSyn preformed fibrils (PFFs) and 6‐hydroxydopamine (6‐OHDA), because aSyn PFFs induce spreading/accumulation of aSyn, and 6‐OHDA rapidly causes local neuronal loss.

**Methods:**

We injected mouse aSyn PFFs into the medial forebrain bundle (MFB) of Sprague–Dawley rats. We investigated spread of pathological aSyn, phosphorylation of aSyn and tau, oxidative stress, synaptic/neuronal loss and cognitive dysfunction 60, 90 and 120 days after injection. Similarly, we injected 6‐OHDA into the MFB and examined the same parameters 1 and 3 weeks after injection.

**Results:**

Following aSyn PFF injection, phosphorylated aSyn was found distant from the injection site in the hippocampus and frontal cortex. However, despite neuron loss being evident close to the site of injection in the substantia nigra at 120 days post injection, there were no other neurodegeneration‐associated features associated with aSyn including synaptic loss. In contrast, 6‐OHDA caused severe neuronal loss in the substantia nigra at 3 weeks post injection that was accompanied by phosphorylation of aSyn and tau, oxidative stress, loss of synaptic proteins, cognitive and motor dysfunction.

**Conclusions:**

Our results demonstrate that spread/replication and slow accumulation of pathological aSyn may not be sufficient to induce neurodegenerative changes. In contrast, oxidative stress responses in addition to aSyn accumulation were associated with other Parkinson's disease (PD)‐associated abnormalities and cognitive dysfunction. Our results may be important when considering why only some PD patients develop dementia.

**Key points**

- Injection of preformed fibril of α‐synuclein into the medial forebrain bundles induced phosphorylated α‐synuclein in the hippocampus and frontal cortex but no obvious neurodegeneration in these two brain regions in 120 days.
- Injection of 6‐hydroxydopamine in the same site induced free radicals, massive neuronal loss in the substantia nigra and phosphorylated α‐synuclein in the hippocampus and frontal cortex with increased oxidative stress in 3 weeks.
- Free radicals seem to be an important factor inducing phosphorylation of α‐synuclein in other brain regions distance from the injection site.

## INTRODUCTION

Abnormal aggregation of beta‐amyloid (Aβ), tau and aSyn are hallmark pathologies in several neurodegenerative disorders, and this trio of proteins has been postulated to act synergistically leading to exacerbated neurodegeneration, behavioural and cognitive impairments [1, 2, 3, 4]. The accumulation and spread and/or templating of pathological proteins may be the first step of sequential neurodegenerative processes, which cause synaptic degeneration/neuronal loss (second step) and impairment of behaviour (e.g., motor or cognitive functions as the third step). Neuroinflammation may promote disease progression throughout this process [5]. We investigated the relationship between these events, with the aim of better understanding the neurodegenerative process involved in the emergence of cognitive dysfunction in models of Parkinson's disease (PD) that could give clues into the mechanisms underlying the conversion of PD to PD dementia (PDD).

PD is characterised by its well‐known neuropathological hallmarks, that is, Lewy bodies (LBs) and Lewy neurites (LNs), that are composed primarily of misfolded aSyn. LBs contain β‐sheet rich aSyn amyloid fibrils, frequently observed across all synucleinopathies [6, 7]. The presence of LBs and LNs also correlates closely with PDD. Approximately 80% of PD patients develop PDD or other cognitive dysfunction 10–15 years after the onset of PD, which leads to the rapid demise of these patients [8]. Unlike AD, where memory loss is predominant, executive functions such as attention, inhibition and visuospatial impairment can be more common than memory loss in PDD [9]. Regions thought to be associated with executive dysfunction and visuospatial impairment include the frontal cortex and hippocampus [10, 11, 12].

The cell‐to‐cell transmission and/or replication of misfolded aSyn are considered key mechanisms in the progression of PD [13, 14]. Dementia in synucleinopathies is thought to be due to aSyn‐linked abnormalities at cortical and hippocampal synapses [15]. Therefore, studying the temporal emergence of aSyn abnormalities allows us to understand the relationship between abnormal forms of aSyn, perturbance of synapses and neuronal loss and cognitive dysfunction.

Although the accumulation of misfolded aSyn is considered by many as key to the pathogenesis of PD and PDD, other mediators such as oxidative/nitrative stress have also been reported to play a role in the neurodegeneration cascade. A bi‐directional relationship between aSyn and oxidative/nitrative stress has been observed in cell culture studies, where each factor can exacerbate the detrimental effects of the other [16, 17, 18]. An increase in oxidative stress markers was also noted in post‐mortem PD brains compared with controls. These markers include 4‐hydroxyl‐2‐nonenal (HNE), a by‐product of lipid peroxidation [19, 20], and 8‐hydroxy‐deoxyguanosine (8OHG), a DNA and RNA oxidation product [21, 22]. Furthermore, the reactive nitrogen species nitrotyrosine is detected in LB's within melanin containing substantia nigra (SN) neurons and other neurons containing amorphous deposits associated with both degenerating and intact neurons, suggesting that oxidative stress is induced in PD [23, 24].

Oxidative stress is also widely believed to be a common mechanism that leads to cellular dysfunction and neurodegeneration in both genetic and idiopathic types of PD [25]. Intracerebral injections of 6‐hydroxydopamine (6‐OHDA) in experimental models of PD trigger oxidative stress responses resulting in the loss of dopaminergic neurons in the SN and dorsal striatum (ST). Dying neurons then produce more reactive oxygen species (ROS) or reactive nitrogen species (RNS) that promote accumulation of pathological proteins [26].

In rodents, injection of aSyn preformed fibrils (PFFs) in the brain induces neuronal accumulation of phosphorylated aSyn which subsequently spreads to interconnected neurons. This accumulation of aSyn is proposed to induce synaptic disruption, neuron loss and cognitive behavioural phenotypes [27, 28, 29]. However, it takes several months after aSyn brain injections before neurodegeneration is apparent [30, 31, 32]. In contrast, injection of the parkinsonian mimetic 6‐OHDA, into the medial forebrain bundle (MFB) region, triggers marked neuronal loss within three weeks [33].

These two experimental models allowed us to investigate the relationship between aSyn spread and neurodegeneration in regions distinct from the site of injection. To achieve this objective, either aSyn PFFs or 6‐OHDA were injected into the MFB of Sprague–Dawley (SD) rats, as this bundle of fibres contains axons that project to the ST, frontal cortex and hippocampus [34]. The MFB is an intricate structure composed of a descending and ascending system of fibres that connect medial and basal forebrain structures [34, 35, 36]. Ascending axons from dopaminergic, serotonergic and noradrenergic neurons located in the lower brain stem also pass through the MFB [34]. The MFB is best known for its connectivity between the ST and SN, making it a popular injection site for investigating PD‐like features in animal models. In rats, the MFB has both output and input projections to and from a diverse range of regions including the locus coeruleus, neocortex, hypothalamus, septum, thalamus and cerebellum [34, 37]. Injection of material into the MFB therefore allows us to study the effects on projection regions such as the frontal cortex that control executive function. Both groups of rats were subjected to behavioural tests to assess cognitive and motor dysfunction, followed by morphological immunohistochemical methods and biochemical analysis to detect specific disease related proteins and abnormal synaptic proteins.

Our results show that neurodegeneration and the onset of cognitive dysfunction do not temporally accompany the progressive accumulation of aSyn in regions distal to the site of PFF injection, including the frontal cortex and hippocampus. However, substantial neuronal loss induced by 6‐OHDA was contemporaneous with aSyn accumulation, synapse loss and cognitive decline. Unlike following PFF injection, a marked oxidative stress response was observed in the 6‐OHDA model. We speculate that oxidative stress exerted directly by 6‐OHDA induces marked neuron loss, with degenerating neurons producing oxidative/nitrative stress environments that facilitates the accumulation of pathological aSyn leading to synapse loss, neurodegeneration and cognitive deficits. Our results provide insights into the mechanisms of PD pathogenesis that may be relevant for determining the rate of conversion of PD to PDD.

## MATERIALS AND METHODS

### Stereotactic injections into the rat MFB

Adult 230–250 g wild‐type male SD rats were obtained from the Center for Comparative Medicine Research, LKS Faculty of Medicine, which is accredited by the Association for Assessment and Accreditation of Laboratory Animal Care (AAALAC). All animal studies were approved by the University of Hong Kong (HKU) and performed in accordance with the Committee on the use of Live animals in teaching and research (CULATR regulations), in which the guidelines of the National Centre for the Replacement, Refinement and Reduction of Animals in Research, UK, and NIH Guidelines for Survival Rodent Surgery, USA are followed.

The rats had constant access to food and water and were housed 2–3 per cage under a 12‐h light/12‐h dark cycle. On the day of surgery, PFFs prepared from recombinant mouse aSyn were thawed and sonicated as previously described [38]. Rats anaesthetised with ketamine hydrochloride (100 mg/kg, intraperitoneally) (i.p.) and xylazine (10 mg/kg, i.p.) were immobilised on a stereotaxic frame (Narishige Scientific Instruments Lab, Japan), adapted from Zhang et al. [39]. Rats were aseptically injected unilaterally in the MFB: AP = −4.0, ML = −1.2 and DV = +7.5 (below dura), from coordinates adapted from Shah et al. and Torres et al. [33, 40] relative to the bregma, either with recombinant aSyn PFFs (6 μg/μl; 30 μg), 6‐OHDA (3 μg/μl; 12 μg) or sterile phosphate‐buffered saline (PBS), 5 μl for the aSyn PFFs group or 4 μl for the 6‐OHDA group, using a 10 μl syringe, gauge 33 (Hamilton, NV) at a rate of 0.5 μl/min. Post‐surgery, rats received Metacam® (Meloxicam, Boehringer, Ingelheim, Germany) at 1 mg/kg into their drinking water as analgesia and were monitored regularly as described elsewhere [33].

### Behavioural testing

Smart 3.0 software (Panlab, USA) was used when applicable in conjunction with video camera recordings to allow the tracking of animal movement during behavioural tests and subsequent analyses. Unless stated otherwise, behavioural experiments were not conducted consecutively within a 2‐day period to ensure that rats had enough rest in between tests. Rats were also habituated inside the behaviour room for 30 min prior to testing. Rats injected with aSyn PFFs and control material were from separate cohorts from those injected with 6‐OHDA and their controls. Rats went through the battery of tests in the same order, that is, open field testing followed by asymmetric cylinder test and concluding with testing in the Morris water maze (MWM).

Locomotion and anxiety‐related activity were assessed using open field testing according to previously published protocols [41]. Rats were examined for asymmetry of forelimb use by the cylinder test as previously described [42]. Analysis of drug‐induced rotations was recorded digitally on a camcorder for 30 min using a previously described protocol adapted from Jerussi and Glick [43]. To measure visuospatial related cognitive impairment, the escape latency and number of platform crossings were recorded using the MWM task. The MWM was used here as previously described [44, 45, 46].

### Collection of tissue

At 60, 90 or 120 d.p.i. of aSyn PFF, in accordance with previous studies [30, 32], and 1 or 3 weeks post‐injection (w.p.i.) of 6‐OHDA [33], rats were anaesthetized with i.p. injections of pentobarbitone at 120 mg/kg followed by transcranial perfusion with cold 0.9% saline and 4% paraformaldehyde (PFA). Brains were dissected and post‐fixed overnight in 4% PFA for histopathology or were snap frozen on dry ice and stored at −80**°**C for biochemistry, as previously described [33, 47].

### Human brain tissue

Formalin‐fixed, paraffin‐embedded brain sections at 7 μm thick from neuropathologically assessed human brain with tau pathology at Braak stage VI (AD; temporal cortex) were obtained as previously described in from Brains for Dementia Research/The London Neurodegenerative Disease Brain Bank, King's College London, UK [48].

### Immunohistochemical staining

Fixed rat brain tissues were embedded into paraffin blocks, sectioned at 7 μm and immunolabelled as previously described using antibodies against pSer129 aSyn (81A, CNDR 1: 10,000), AT8 (Thermo scientific #MN1020, 1:50), PHF1 (Peter Davies 1:100), tau pS404 (Thermo scientific #44‐758G, 1:20), 8‐hydroxy‐2‐deoxyguanosine (Abcam #ab62623, 1:150) and anti‐nitrotyrosine antibody (Thermo scientific #A‐21285, 1:500) [47].

### Western blot

Western blot was performed following determination of sample protein concentration using a BCA assay kit (Pierce, Thermo; Rockford, IL, USA). Membranes were probed with antibodies against glial fibrillary acidic protein (DAKO/Agilent Technologies, 1:1000), synaptophysin (Santa Cruz, #sc‐17750, 1:1000), synaptotagmin (Abcam, #ab51164 1:1000), PSD‐95 (CST, #3450, 1:1000), NR2B (Millipore, #06‐600, 1:1000) and β‐actin (Abcam, #ab8227, 1:5000).

### Quantification and statistical analysis

All statistical analyses were performed on Prism V6.0 (GraphPad Software, Inc., USA). Figure legends report the statistical analysis performed, the number of rats (*n*) analysed in each experiment and the *p* values. The level of significance was set at *p* < 0.05. All data are presented as mean ± SEM.

## RESULTS

### aSyn PFFs induced accumulation of phosphorylated aSyn but not phosphorylation of tau in the frontal cortex and hippocampus

**FIGURE 1:**
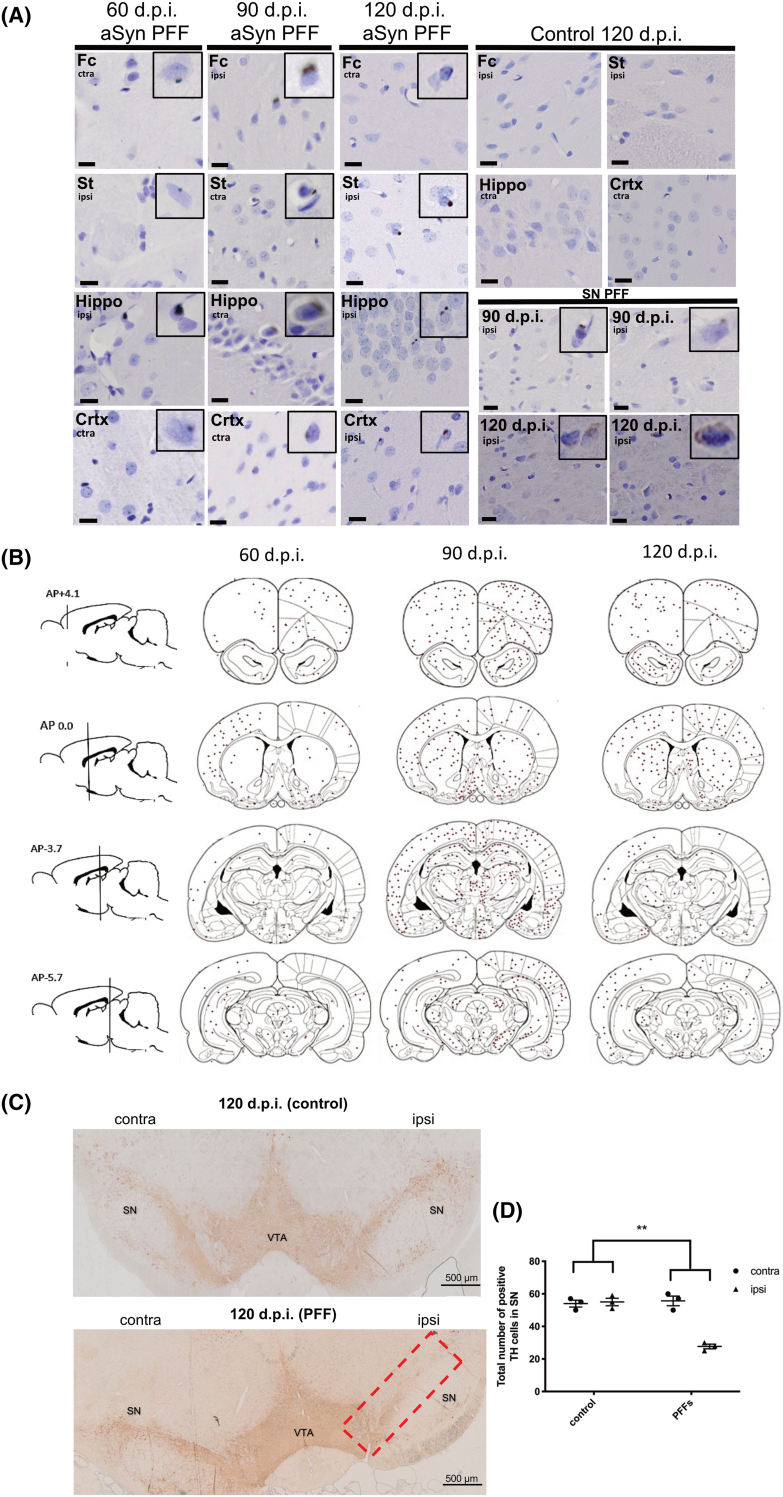
Phosphorylated alpha‐synuclein (aSyn) slowly accumulates in regions distant from the preformed fibril (PFF) injection site. (A) Using a stereological approach, a 1 in 20 sequence of 7 μm coronal sections was taken throughout the entire brain (both contralateral and ipsilateral hemispheres) of animals injected with aSyn PFFs for 60, 90 and 120 d.p.i. or control (vehicle) for 120 d.p.i. Representative images are shown. Sections were immunolabelled with an antibody against aSyn phosphorylated at Ser129 and counterstained with haematoxylin to label nuclei. Phosphorylated aSyn inclusions appeared as faint cytoplasmic and denser perinuclear inclusions in the frontal cortex (Fc), striatum (St), hippocampus (Hippo), cortex (Crtx) and substantia nigra (SN). Control animals did not show any positive labelling. Scale bars = 10 μm, *n* = 3. (B) The total number of positively immunolabelled cells in three animals from each PFF‐injected group was counted and the positions marked on coronal brain tracings. Cells positive for phosphorylated aSyn were observed in many regions including those directly and indirectly connected to the medial forebrain bundle (MFB) and appeared to peak at 90 d.p.i. The red circle indicates the site of injection (MFB). Regions proximal to the injection site appeared to show the most abundant phosphorylated aSyn positive cells; (C) 7 μm coronal sections from control and PFF‐injected rats at 120 d.p.i. were immunolabelled using an antibody raised against tyrosine hydroxylase (TH). Representative images are shown. Red box identifies region of interest used for quantification. (D) Quantification of TH cell count for 120 d.p.i. tissues. To detect differences in cell number between PFF and control groups, an unpaired *t* test was used. This showed a statistically significant reduction in TH cell number in the PFF group. **p < 0.01. Data shown are mean ± SEM, *n* = 3.

The spread and/or replication of phosphorylated aSyn after aSyn PFF injections into various anatomical regions has been widely demonstrated [28, 29, 31, 32, 49]. Here, we show that injection of aSyn PFFs into the MFB of SD rats (Figure S1) resulted in phosphorylated aSyn immunoreactivity being detected in the frontal cortex, hippocampus, ST and SN (Figure 1A) from 60 d.p.i. Phosphorylated aSyn was detected in both hemispheres, with the number of positively labelled cells peaking at 90 d.p.i. as indicated by the load of brown circles, each of which represents a positively immunolabelled neuron (Figure 1B). No phosphorylated aSyn was found in the brain of PBS‐injected controls. Quantification of the total positive immunolabelled phosphorylated aSyn count within each region for all time points is shown in Figure S2. Although immunoreactivity for phosphorylated aSyn can often be indicative of aSyn aggregates, we further investigated the solubility of aSyn in frontal cortex samples taken at 90 d.p.i. on the basis of its solubility in increasingly stringent detergents, as has been performed previously [7]. We were unable to detect any aSyn that was insoluble in sarkosyl, with the majority of aSyn being isolated in high salt, high salt triton‐x and sarkosyl‐soluble fractions (Figure S3). This suggests that the aSyn we detected by immunohistochemistry (IHC) may not be bona fide aggregates, at least on the basis of their solubility in these detergents. It is possible, however, that the relative sparsity of aSyn inclusions prevents us from readily detecting aggregates in whole tissue homogenates.

Although there is abundant evidence of a synergistic relationship between aSyn and tau in LB disorders (LBDs) such as PD [3, 50, 51], aSyn PFFs per se rarely lead to any change in tau [52], with only specific strains capable of inducing tau phosphorylation [53, 54]. We performed immunohistochemical staining for tau phosphorylated at S404 and pS396/404 (PHF1). No substantial immunoreactivity was noted for any of these epitopes within the frontal cortex or hippocampus of either aSyn PFF or sham‐injected groups at 90 d.p.i. (Figure S4). With the use of a similar approach to that described for aSyn, we also found no apparent differences in the solubility of tau between control‐ and PFF‐injected rats (Figure S5).

aSyn did not induce any further neurodegeneration‐associated changes in the frontal cortex or hippocampus, although significant neuronal loss was observed in the SN from 120 d.p.i.

To determine if any common features of PD‐like neurodegeneration resulted from aSyn PFF injection into rat MFB, we examined synaptic protein levels. It has previously been reported that aSyn PPFs are able to compromise synaptic activity and enhance synapse loss in cultured neurons [55]. Western blots showed no significant alteration in the levels of the post‐synaptic proteins PSD‐95 and NR2B or the presynaptic proteins synaptotagmin and synaptophysin in either frontal cortex or hippocampus of aSyn PFF‐injected rats at 90 d.p.i relative to control (Figure S6).

Previous work has demonstrated progressive dopaminergic neuron loss within the SN upon administration of aSyn PFFs [28, 32, 56, 57]. Of the three time points being investigated in this study, a statistically significant TH cell loss was noted at 120 d.p.i. (Figure 1C,D) but not at earlier time points (data not shown). Despite this neuronal loss, there was no evidence of other neurodegenerative changes in these samples or neuronal loss in other brain regions by 120 d.p.i.

### The Parkinsonian mimetic 6‐OHDA induced phosphorylation of aSyn and tau in the frontal cortex and hippocampus

**FIGURE 2:**
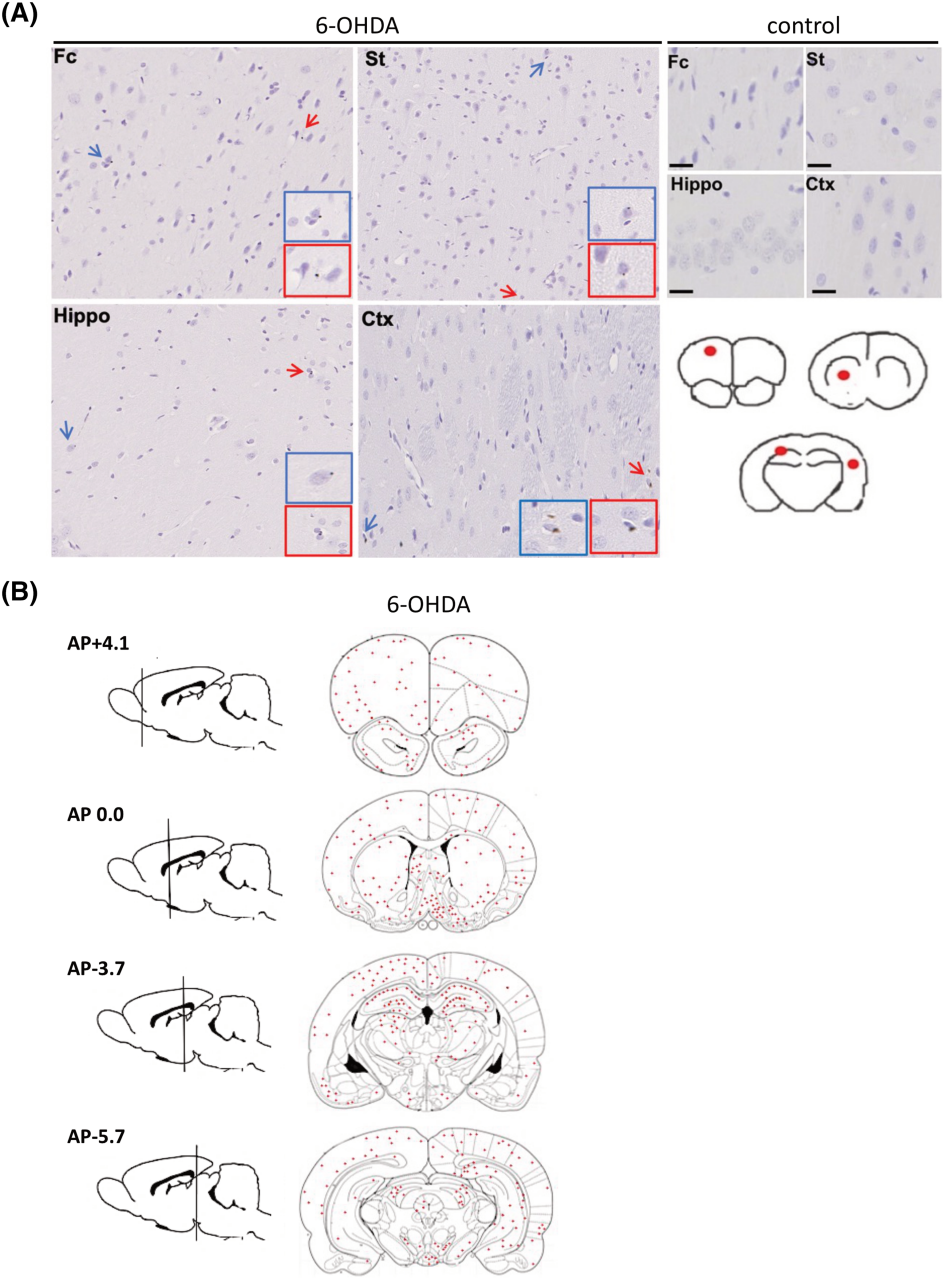
Phosphorylated alpha‐synuclein (aSyn) rapidly accumulates in regions distant from the 6‐OHDA injection site. (A) Immunohistochemistry was performed on 7 μm thick brain tissues from rats unilaterally injected with phosphate‐buffered saline (PBS) (control) or 6‐OHDA into the medial forebrain bundle (MFB). An antibody raised against phosphorylated aSyn at serine 129 (81A) was used to label cells containing phosphorylated aSyn. Control rats (top right panel) did not show any 81A immunoreactivity, whereas cells showed faint positive labelling for phosphorylated aSyn in Fc, St, Hippo and Crtx in response to 6OHDA (middle panel). Red scale bar 20 μm, black scale bar, 5 μm, *n* = 3. Blue and red arrows indicate cells immunoreactive to the phosphorylated aSyn antibody, with coloured insets showed higher magnification images of these cells. Images are from representative rodents. Fc = frontal cortex, St = striatum, Hippo = hippocampus and Crtx = cortex. Red circles on brain traces (bottom right panel) show the Fc, striatum, hippocampus and cortical areas from which sections were obtained and immunolabelled. (B) The total number of positively immunolabelled cells in three animals from the 6‐OHDA‐injected group was counted and the positions marked on coronal brain tracings. Cells positive for phosphorylated aSyn were observed in many regions including those directly and indirectly connected to the MFB.

Our results showed that the appearance of pathological aSyn in regions distant from the MFB requires extended time (over 120 d.p.i.), despite neuronal loss in the SN being detected at 120 d.p.i. To further understand the neurodegenerative processes, we injected the Parkinsonian mimetic 6‐OHDA into the MFB of another cohort of rats. Three weeks post‐injection, we performed immunohistochemical staining on brain sections from 6‐OHDA‐injected rats and their controls. We observed some phosphorylated aSyn‐positive cells in the frontal cortex and hippocampus of 6‐OHDA‐injected rats but not in PBS‐injected controls (Figure 2A). Individual immunoreactive neurons in the frontal cortex, ST, hippocampus and other cortical areas are represented in Figure 2B. The total number of positively immunolabelled phosphorylated aSyn cells within each of those regions is shown in Figure S7. aSyn immunoreactivity was not observed in these regions at 1 week post‐injection (Figure S8). These findings show that 6‐OHDA can induce modest phosphorylation of aSyn in brain regions distal from the site injection in a rapid time frame (3 weeks).

**FIGURE 3:**
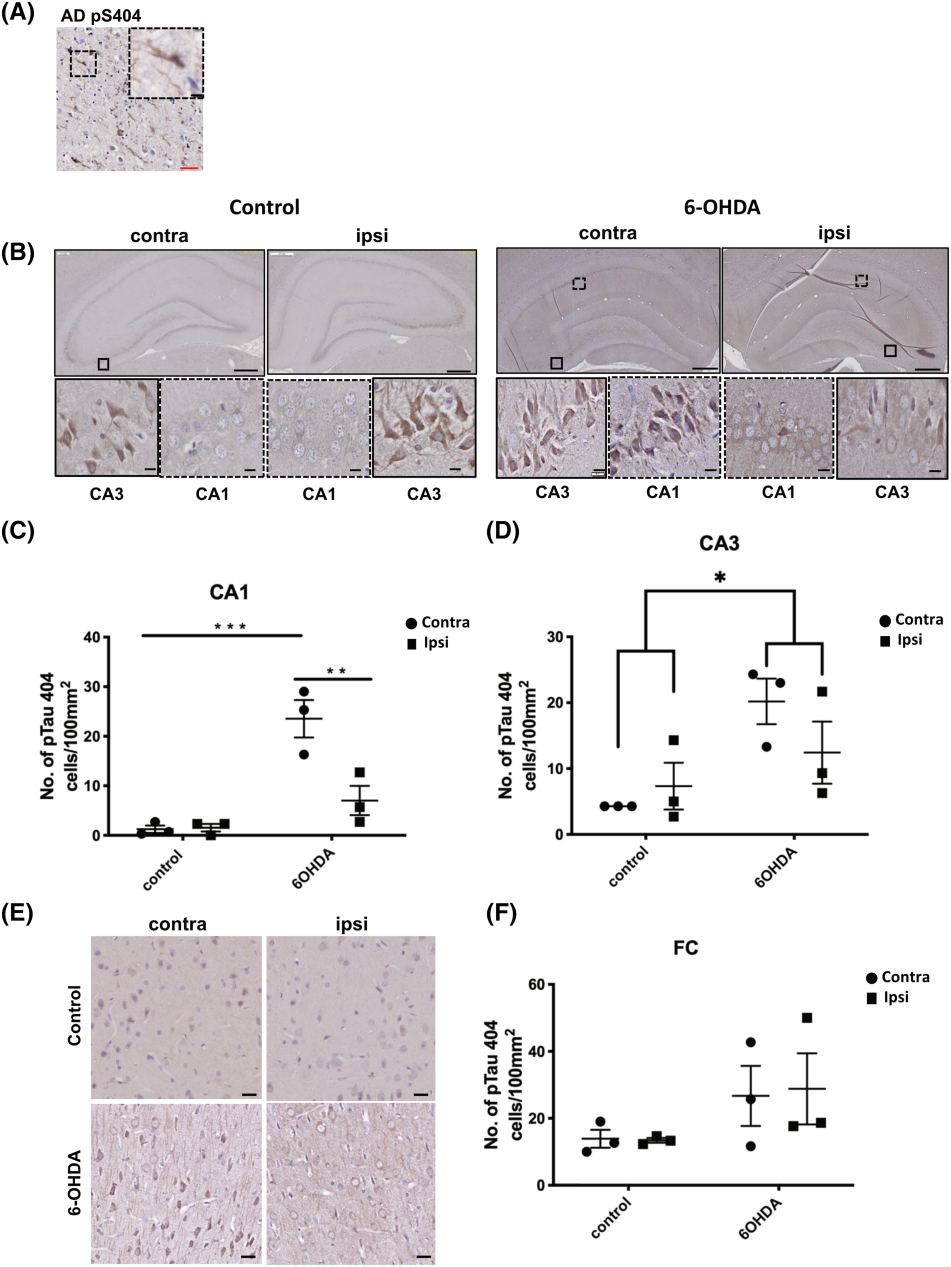
(A) Tau phosphorylated at Ser404 rapidly accumulates in regions distant from the 6‐OHDA injection site. Immunohistochemical staining was performed on 7 μm paraffin embedded sections from the temporal cortex of a post‐mortem Alzheimer's disease (AD) brain using an antibody against pTau404. Inset shows higher magnification of immunoreactive neurons. For rat brain, 7 μm paraffin embedded sections were labelled with the same antibody against pTau404. (B) Representative images of pTau404 immunolabelling at 3 weeks post injection. Sections were counterstained with haematoxylin. Both control and 6‐OHDA‐injected animals showed labelling of pTau404 in CA3. pTau404 labelling was also apparent in CA1 of 6‐OHDA animals but not control animals. Bar charts in show quantification of the number of pTau404 +ve cells in (C) CA1 and (D) CA3. (E) Representative images of pTau404 immunolabelling in frontal cortex (Fc). Sections were counterstained with haematoxylin. Bar chart in (F) shows the number of pTau404 +ve cells in frontal cortex. Scale bars in main images are 500 μm, and 5 μm scale bars are used in insets. Unpaired *t* tests were used to determine differences in the number of immunoreactive cells between control and 6‐OHDA groups for either the contralateral or ipsilateral hemisphere and demonstrated a statistically significant increase in pTau404‐immunoreactive cells in the CA1 and CA3. No differences were found for the Fc region. *p < 0.05, ***p < 0.001. Data shown are mean ± SEM, *n* = 3.

**FIGURE 4:**
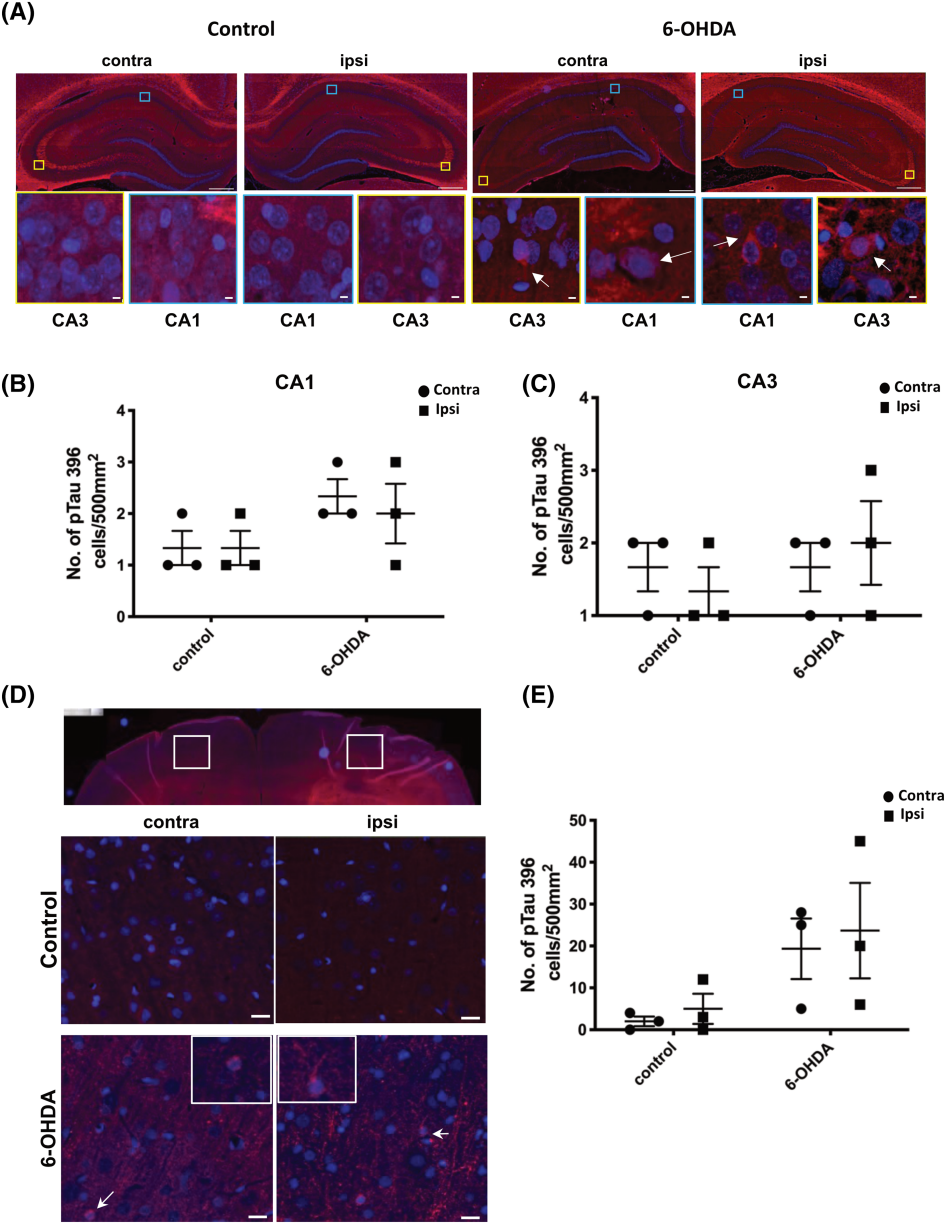
Cytoplasmic inclusions of tau phosphorylated at Ser396 are apparent following 6‐OHDA injection into the medial forebrain bundle (MFB). (A) Representative immunofluorescence labelling of pTau396 in frontal cortex areas shown in low magnification images with white boxes, at 3 weeks post injection. DAPI (blue) used to stain nuclei. Control rat brain showed some background labelling, but unlike 6‐OHDA‐treated rats, little cytoplasmic pTau396 immunoreactivity was observed. Yellow and blue boxes indicate regions of CA3 and CA1, respectively, that are shown at higher magnification in insets. White arrows indicate specific areas of positive tau immunoreactivity in 6‐OHDA‐injected rat tissues. Scale bars in the main images are 500 μm, and 5 μm scale bars are used in the insets. Graphs show the number of pTau396 positive cells per mm^2^ in the contralateral (contra) and ipsilateral (ipsi) hemispheres for (B) CA1 and (C) CA3. (D) Representative images of pTau396 immunolabelling in the frontal cortex (Fc). White arrows indicate area shown at higher magnification in insets. Scale bar 20 μm. (E) Quantification of the number of pTau396 positive cells in Fc. Unpaired *t* tests were used to determine differences in the number of immunoreactive cells between control and 6‐OHDA groups for either the contralateral or ipsilateral hemisphere and did not indicate any statistical significance. Data shown are mean ± SEM, *n* = 3.

We also examined tau phosphorylation in the frontal cortex and hippocampus. The phosphorylation and synaptic mislocalisation of tau are biological determinants of dementia in AD [58], and modified tau has been reported in PD and PDD brains [3, 4, 59]. Following 6‐OHDA injections, there was increased tau phosphorylation at S404, S396 and S396/404 (PHF1), sites known to be aberrantly phosphorylated in AD brain [60]. Phosphorylation of tau at S404 was found at basal levels in control human brain and is increased in AD [61] (Figure 3A). Accordingly, pS404 and pS396 immunoreactivity was apparent in CA1 and particularly in CA3 of both control and 6‐OHDA‐injected rats (Figures 3B and 4A–C), and labelling intensity was significantly increased in hippocampal sub‐fields in the 6‐OHDA‐injected group (Figure 3C,D). The MFB has indirect projections to the frontal cortex, and this area also displayed immunoreactivity for phosphorylated tau at pS404, particularly in 6‐OHDA‐injected rats, in both contralateral and ipsilateral hemisphere (Figure 3E,F). We also observed pS396 immunolabelling on both contralateral and ipsilateral sides of the frontal cortex, with an apparent increase of pS396 immunoreactivity in the 6‐OHDA‐injected group compared to controls (Figure 4D,E). Immunoreactivity against the PHF1 antibody requires tau phosphorylation at both Ser396 and Ser404 and is characteristic of abnormally modified tau in disease [61] (Figure S9A). Faint PHF1 immunoreactivity was observed in the hippocampus of 6‐OHDA‐injected rats and was largely absent from control rat hippocampus (Figure S9B–E). PHF1 immunoreactivity was mostly absent within the frontal cortex of both control and 6‐OHDA‐injected rats (Figure S9F,G). These results show that 6‐OHDA induces phosphorylation of some tau epitopes that allow tau pathology to be established in the frontal cortex and hippocampus, regions distal to the injection site.

### 6‐OHDA induced loss of the post‐synaptic protein PSD‐95 in the frontal cortex and substantial neuronal loss in the SN

**FIGURE 5:**
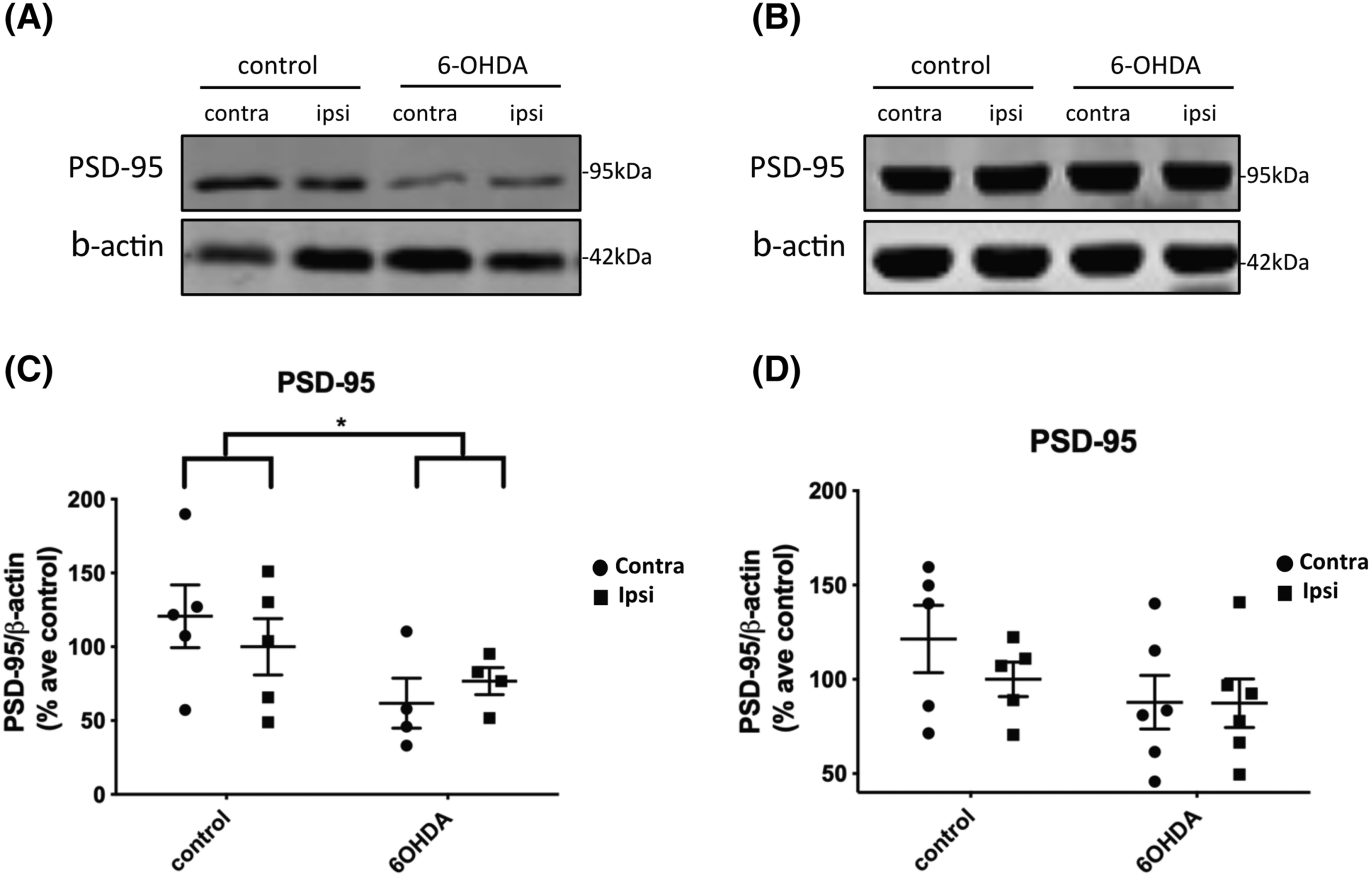
6‐OHDA injection caused a loss of PSD‐95 in the frontal cortex but not hippocampus. (A) Immunoblots of post‐synaptic density protein‐95 (PSD‐95) in the high salt fraction of frontal cortex from 6‐OHDA‐ and control‐injected rats, 3 weeks post‐injection. (A) Representative immunoblots from (A) frontal cortex and (B) hippocampus, probed with an antibody against the post‐synaptic marker, PSD‐95 (95 kDa). β‐Actin was used as a loading control (42 kDa). (C) The dot plot shows a marked reduction in the amount of PSD‐95 relative to β‐actin in frontal cortex of 6‐OHDA‐injected rats relative to controls. Control, *n* = 5, preformed fibril (PFF), *n* = 4. (D) The dot plot shows no significant difference as a result of treatment or between hemispheres in the hippocampus, control, *n* = 5, PFF, *n* = 6. Unpaired *t* tests were used to determine differences in the amount of PSD‐95 between control and 6‐OHDA groups for either the contralateral or ipsilateral hemisphere and showed a significant effect of treatment. *p < 0.05. Data are mean ± SEM presented as % average control where control is the ipsilateral hemisphere of control‐injected rats*.*

Synaptic deficits underlie cognitive decline and dementia in neurodegenerative diseases [15, 62, 63]. Thus, we examined if 6‐OHDA affects the levels of synaptic proteins. Western blot of frontal cortex lysates showed a significant reduction of the post‐synaptic protein PSD‐95 bilaterally (Figure 5A,C). Reduction in PSD‐95 was, however, not noted within the hippocampus (Figure 5B,D) of 6‐OHDA‐injected rats. There were no significant changes in the amounts of NR2B or the pre‐synaptic markers synaptotagmin and synaptophysin in the frontal cortex or hippocampus at 3 weeks post‐injection (Figure S10).

**FIGURE 6:**
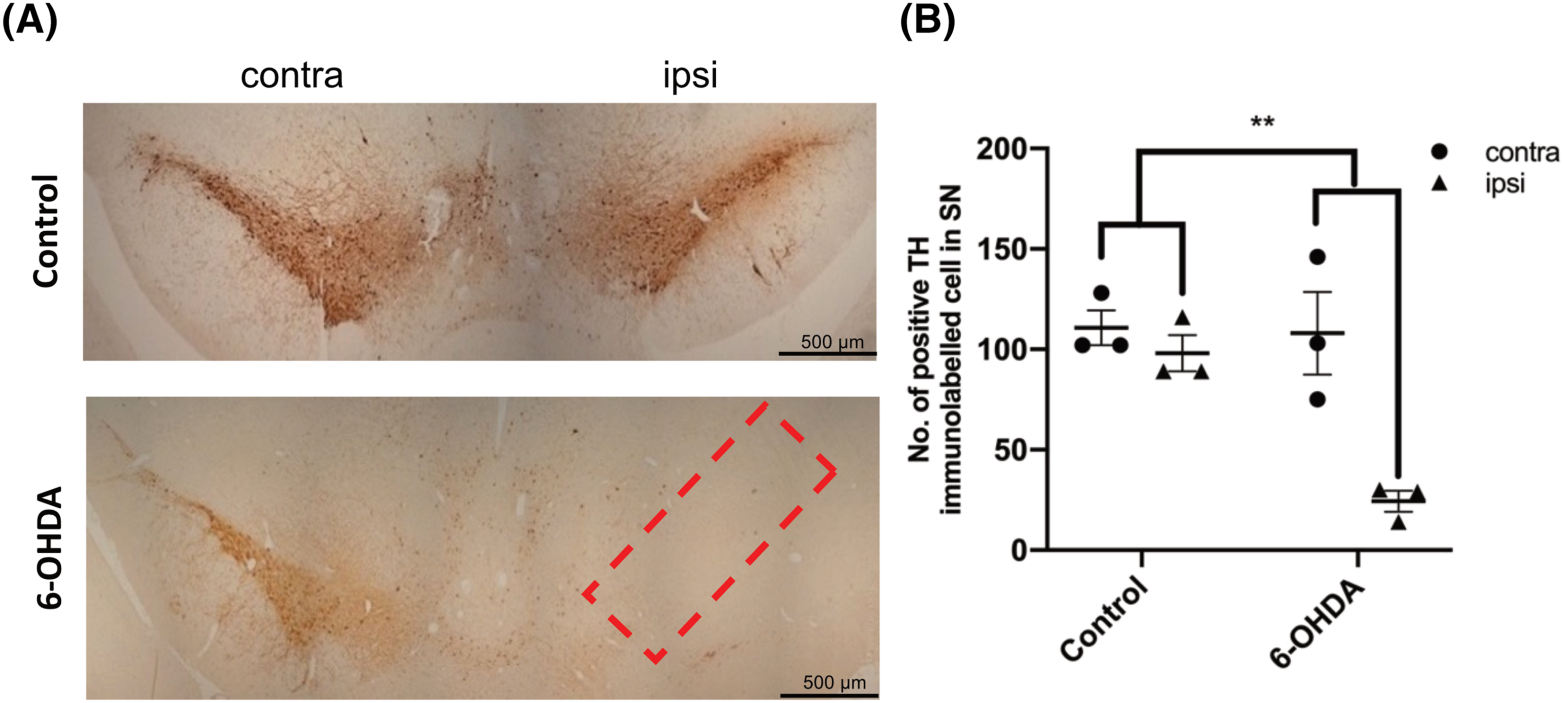
6‐OHDA caused an ipsilateral loss of TH neurons in the substantia nigra 3 weeks after injection; (A) 7 μm sections from control and 6‐OHDA‐injected rats, 3 weeks post‐injection, were immunolabelled using an antibody raised against TH. 6‐OHDA‐injected animals demonstrated an ipsilateral loss of TH +ve cells in the substantia nigra compared with control animals. Red box indicates the region of interest from which TH positive cells were counted. (B) Graph shows quantification of the number of TH positive cells in control and 6‐OHDA animals, for both contralateral and ipsilateral hemispheres. Unpaired *t* tests were used to determine differences in cell count between control and 6‐OHDA groups for either the contralateral or ipsilateral hemisphere and demonstrated a substantial reduction in TH positive cells within the ipsilateral hemisphere of 6‐OHDA rats compared with control. **p < 0.01. Data are mean ± SEM, *n* = 3.

We also examined neuronal loss in the SN as this is a defining feature of PD and PDD. 6‐OHDA‐injected rats showed substantial loss of TH‐positive neurons in the ipsilateral SN at 3 weeks post‐injection (Figure 6A). A statistically significant reduction in TH immunolabelling was also noted between the ipsilateral hemisphere of control rats and both hemispheres of 6‐OHDA‐injected rats after 3 weeks (Figure 6B). Because dying neurons release several toxic factors that may be transmitted to other regions of the brain along projections, SN neuron loss may influence the rapid neurodegenerative changes we found in the frontal cortex and hippocampus.

### Oxidative and nitrative stress was apparent in the frontal cortex and hippocampus following 6‐OHDA injection into the MFB

**FIGURE 7:**
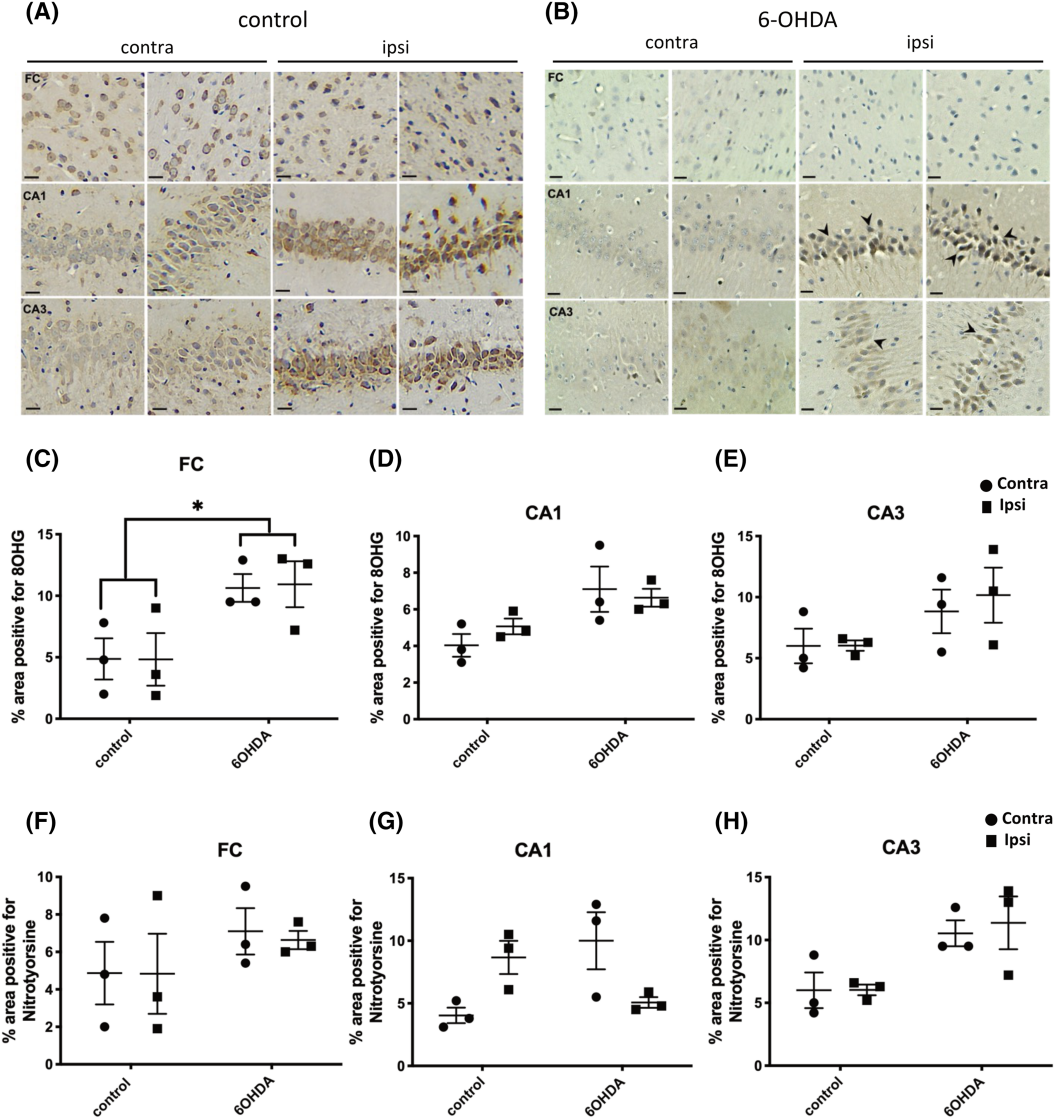
6‐OHDA injection induced a widespread oxidative stress response; 7 μm sections from control and 6‐OHDA‐injected rats, 3 weeks post‐injection, were immunolabelled using antibodies raised against (A) anti‐8‐hydroxyguanosine (8OHG) to detect damage in DNA/RNA and (B) nitrotyrosine to indicate nitrative damage. Regions of the brain shown include frontal cortex and hippocampal subfields, CA1 and CA3, both contralateral (contra) and ipsilateral (ipsi) hemispheres. Scale bar 20 μm. Graph shows quantification of the % area positive for 8OHG in (C) frontal cortex (Fc), (D) CA1 and (E) CA3, and for nitrotyrosine in (F) Fc, (G) CA1 and (H) CA3. Unpaired *t* tests were used to determine differences in immunoreactivity between control and 6‐OHDA groups for either the contralateral or ipsilateral hemisphere and showed a statistically significant increase in 8OHG in the Fc. No other differences between groups were found. *p < 0.05. Data are mean ± SEM, *n* = 3.

One type of toxic factor released from dying neurons are free radicals, which trigger both oxidative stress and nitrative stress. Therefore, we examined oxidative stress in the frontal cortex and the hippocampus 3 weeks post‐injection by immunostaining with an antibody against 8OHG, and nitrative stress was assessed by immunoreactivity to anti‐nitrotyrosine antibody. Increased oxidative stress responses in the frontal cortex were confirmed using an antibody specific to 8OHG (Figure 7A,C) within the 6‐OHDA‐injected group compared with control rats. Although the hippocampal subfields CA1 and CA3 also demonstrated an apparent increase in immunolabelling, it was not significant (Figure 7D,E). We found no significant increase in the percentage of 8OHG‐positive cells in the frontal cortex, hippocampal CA1 or CA3 regions of aSyn PFF rats at 90 d.p.i (Figure S11B–D).

With the use of anti‐nitrotyrosine antibodies (Figure 7B), we also noted a non‐significant increase in nitrotyrosine‐positive cells within the frontal cortex and hippocampal regions in 6‐OHDA‐injected rat brains when compared with control brains (Figure 7F–H). Similar to the results from 8OHG immunolabelling, aSyn PFFs rats did not show a marked increase in nitrotyrosine‐positive cells within the frontal cortex and hippocampal regions compared with control rats (Figure S11E).

To further explore the timing of these events, we also examined changes in rats 1 week following injection of 6‐OHDA. At this timepoint, there is no significant loss of TH +ve cells in the SN, although there was a trend towards a reduction in cell number in the contralateral hemisphere of 6‐OHDA rats relative to controls (Figure S12). Oxidative stress was found to be significantly elevated in the SN, but not other regions, 1 week after 6‐OHDA injection (Figure S13). This may result from the close proximity of the SN and MFB injection site.

Taken together, these data may suggest that in the absence of an oxidative stress response, aSyn PFFs may not be sufficient to induce rapid and major neurodegenerative changes in the frontal cortex and the hippocampus, despite the presence of phosphorylated aSyn.

### 6‐OHDA‐injected rats exhibited deficits in hippocampal‐dependent visuospatial learning and an asymmetric motor impairment

**FIGURE 8:**
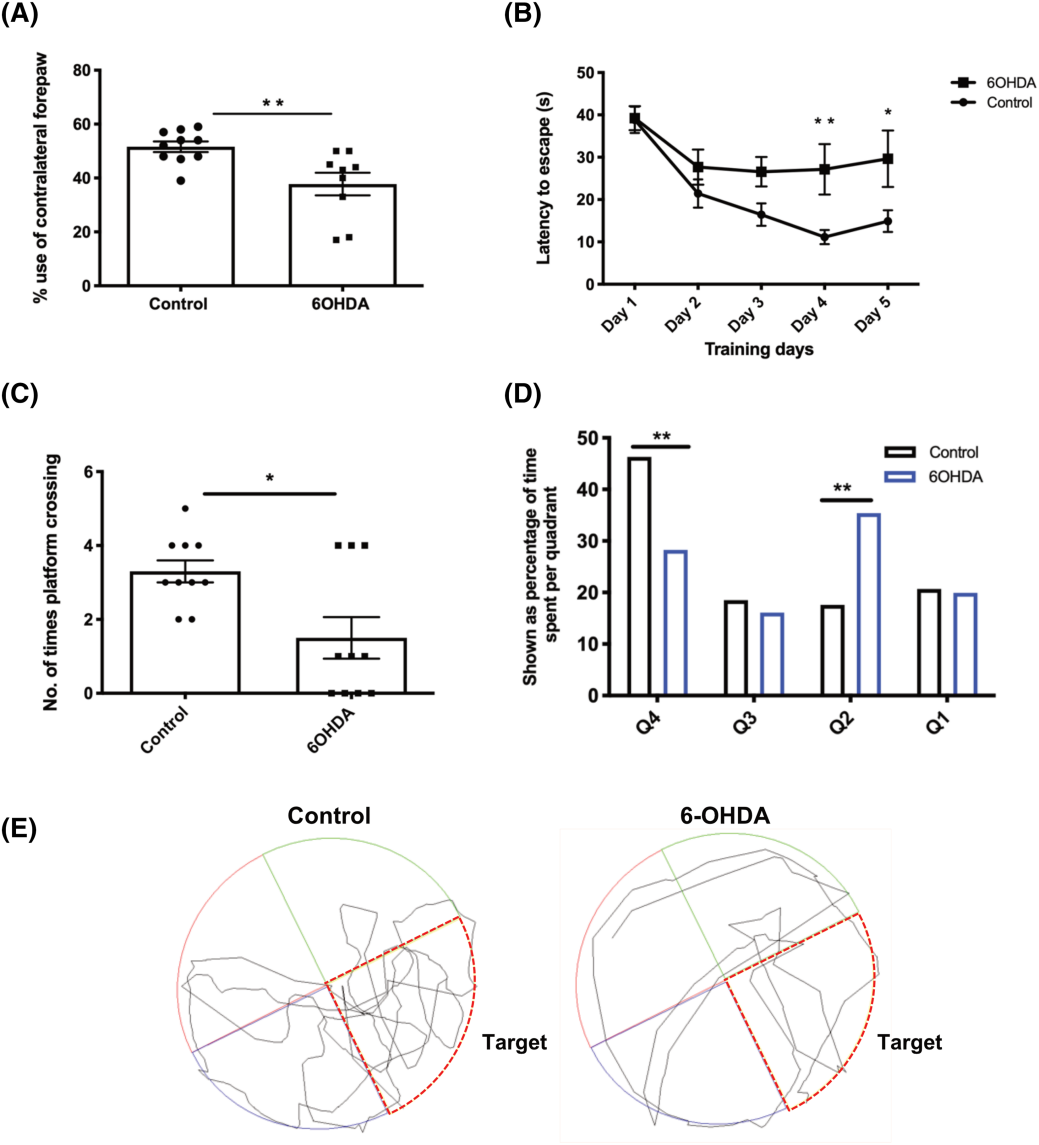
6‐OHDA injection into rat medial forebrain bundle (MFB) induces motor and cognitive impairments. (A) The graph shows that 6‐OHDA‐injected animals show a contralateral forepaw impairment, with significantly reduced use of the contralateral forepaw compared with control animals during the asymmetric cylinder test, signifying a fine motor deficit. Data are mean ± SEM, control, *n* = 10, 6‐OHDA, *n* = 9. **p < 0.01, statistical test used, unpaired *t* test. (B) Graph shows the learning curve during training Days 1–5. 6‐OHDA‐injected rats showed a learning deficit throughout the task compared with control animals where there was a reduction in escape latency from the initial training day. 6‐OHDA‐injected rats took significantly longer than control rats to escape the Morris water maze (MWM) on Days 4 and 5. Analysis with repeated 2‐way analysis of variance (ANOVA) indicated a significant effect of treatment on escape latency but no effect of timepoint or a timepoint × treatment interaction. Post hoc analysis by Tukey's multiple comparisons test showed significant differences between the control and 6‐OHDA‐treated groups on the 4th and 5th days of training. *p < 0.05, **p < 0.01. Data are mean ± SEM, *n* = 10. (C) The number of platform crossings during the probe trial indicated that 6‐OHDA‐injected rats show a deficit in reference memory compared with controls with fewer numbers of platform crossings. (D) Percentage of time spent per quadrant during the probe test. Analysis with repeated 2‐way ANOVA indicated a significant effect of treatment on quadrant preference but no effect of timepoint or a timepoint × treatment interaction. Post hoc analysis by Šidáks multiple comparisons test showed a significant effect of treatment for this parameter. **p < 0.01. Data are mean ± SEM, *n* = 10. (E) Traces of swim trajectories in the MWM probe test. Control rats, but not 6‐OHDA‐injected rats, show clear preference for the target quadrant (red outline).

It is established that 6‐OHDA induces balance and/or coordination disabilities [64, 65]. Here, the asymmetric cylinder test was used to detect motor asymmetry. We observed a unilateral motor deficit with a reduction in forepaw usage during the asymmetric cylinder task in rats injected with 6‐OHDA compared with control rats (Figure 8A). It is well‐known that motor dysfunction is largely correlated with neuronal loss in the SN. However, in aSyn PFF‐injected rats, we found only a transient reduction in contralateral forepaw usage at 90 d.p.i. (Figure S14A) but not at 60 d.p.i. or 120 d.p.i. (data not shown). These results suggest that there was no severe impairment of motor function in the aSyn PFF‐injected rats, most likely because there was no significant loss of neurons in the SN following injection of aSyn PFF into rat MFB.

Lastly, to understand whether the neurodegenerative changes we observed in frontal cortex and hippocampus in 6‐OHDA‐injected rats caused cognitive deficits, the MWM was used to assess visuospatial impairment [45] by adapting a protocol previously established for this purpose [46]. Three weeks following injection of 6‐OHDA, rats displayed an impairment during the training phases of the MWM, and their escape latencies in comparison with control rats were significantly increased (Figure 8B). On Day 1, both control and 6‐OHDA groups showed the same escape latency. However, as the training phase proceeded, it became evident that 6‐OHDA‐injected rats were impaired whereas the control rats had a reduced escape latency time demonstrating learning. Furthermore, by Day 3, both groups plateaued in their learning, with control groups having a shorter escape latency overall, compared with 6‐OHDA‐injected rats. In addition to the spatial training phase, the MWM also detects reference memory. 6‐OHDA‐injected rats demonstrated an impairment in reference memory shown by decreased performance in locating the previous position of the platform in the probe trial (Figure 8C,D). Although analysis of the swim distance showed a significant difference between control and 6‐OHDA‐injected animals (Figure S15), the swim trajectories showed that control, but not 6‐OHDA‐injected rats, showed an evident preference for the target quadrant, which despite 6‐OHDA‐injected rats showing reduced swim length and speed demonstrates cognitive impairment in the 6‐OHDA‐injected animals (Figure 8E).

In contrast, despite the presence of phosphorylated aSyn labelling in neurons in the brains of the aSyn PFF‐injected rats, we did not observe any sustained differences in cognition between control and aSyn PFF groups. Rats underwent behavioural tests as previously described for 6‐OHDA‐injected rats to detect any impairments in visuospatial activity and motor function at 60, 90 and 120 d.p.i. We found no difference in any MWM parameters examined between control and aSyn PFF‐injected groups at any of the time points investigated (data shown from 90 d.p.i. only in Figure S14B–D).

### 6‐OHDA and aSyn‐PFF‐injected rats did not exhibit anxiety behaviour

Finally, to ensure that rats did not exhibit any anxiety, which could affect their behavioural outcomes, we used the open field task to monitor anxiety related behaviour. Neither control nor 6‐OHDA rats exhibited anxiety behaviour (Figure S16B), in keeping with the findings reported by others [66]. aSyn PFF‐injected rats and controls also did not demonstrate anxiety behaviour. All groups spent similar amounts of time in the open centre zone (inner zone) (Figure S16A,B). Next, we investigated the rodents' land‐based locomotor activity. Although there was a slight reduction in the distance travelled, there was no overall significant difference between control and 6‐OHDA‐injected groups or control and PFF groups (Figure S16C,D). Trajectories of representative rats during the open field task are shown (Figure S16E,F).

## DISCUSSION

It is commonly considered that a neurodegenerative cascade is initiated downstream of the accumulation of aggregated proteins, eventually leading to degeneration of synapses and neurons, behavioural and/or cognitive deficits. Neuroinflammation can occur throughout disease course. Our study showed that aSyn PFFs induced the progressive appearance of phosphorylated aSyn throughout several regions of brain that are consistent with the concept of aSyn spread. This is in agreement with numerous reports showing that intracerebral injections of aSyn PFFs induce aSyn propagation in mice and rats [28, 29, 31, 32, 49]. The phosphorylation and accumulation of aSyn can affect synapses and mitochondria and are detrimental to neuron health [67]. However, we find that neurodegenerative events associated with phosphorylated aSyn following aSyn PFF injection are relatively slow to emerge. Similar findings were recently reported by others [5]. In contrast, 6‐OHDA injection into the MFB caused rapid and marked neuronal death in the SN. This was associated with oxidative/nitrative stress in connected brain regions from as early as 1 week post‐injection. There is a strong association between oxidative stress and aSyn accumulation [16, 68, 69], and in‐keeping with this, we found accumulations of phosphorylated aSyn and tau in the frontal cortex and hippocampus, in addition to perturbation of synaptic proteins. Our study strongly suggests that increased levels of oxidative/nitrative stress as a result of neurotoxic insult rapidly progressed to neurodegeneration in distal brain regions. We propose that such oxidative stress responses could be an important factor in the rapid conversion of PD to PDD.

It is believed that dementia associated with synucleinopathies results from a loss of connectivity within neuronal circuits [70] through which aSyn can spread [70, 71]. The MFB has traditionally been considered an ideal injection site for modelling PD because of its key projections to areas commonly associated with PD including the ST and SN. Furthermore, the MFB also has afferent and efferent projections to regions associated with executive function and visuospatial impairment in PD. Findings from animal studies have demonstrated that the spread/propagation of aSyn and tau pathology is determined by neuroconnectivity and not proximity [14, 72]. Therefore, aSyn PFFs were injected into the MFB to allow investigation of the role of aSyn in contributing to cognitive deficits in rats. We demonstrated that intracerebral injections of aSyn PFF into the rat MFB triggered trans‐neuronal spreading of aSyn as indicated by the accumulation of phosphorylated aSyn in connected regions [14, 31, 32, 57]. Similarly, phosphorylated aSyn was found in the frontal cortex and hippocampus following 6‐OHDA injection into rat MFB. While the vast majority of studies target major terminal fields [28, 30, 32, 57], the MFB injections are directed towards fibre‐mediated effects, and the results suggest that neural projections of the MFB can promote progressive regional neurodegeneration. However, we were unable to detect any accumulation of sarkosyl‐insoluble aSyn in these models. While it is possible that our methods were not sufficiently sensitive to detect aSyn aggregates, or soluble replication‐competent aSyn oligomers, these data raise the possibility that the progressive emergence of phosphorylated aSyn is secondary to downstream changes resulting from local effects of aSyn PFF or 6‐OHDA, rather than being the result of aSyn spread.

Although the characteristic hallmarks of PD are LBs and LNs, around 80% of patients show coexistent AD‐like pathology [59, 60, 73, 74, 75, 76]. Cross‐seeding of tau and aSyn has previously been demonstrated [27], and tau is closely linked with synaptic degeneration in dementia [58, 77]. We have used wild‐type rats in this study, and these animals do not readily produce Aβ aggregates and so they would not be expected to produce any Aβ pathology even following aSyn PFF injection. Indeed, this has been recently reported by others [52]. Therefore, we investigated if tau phosphorylation was induced by 6‐OHDA. Accordingly, we found an increase in tau phosphorylation in our 6‐OHDA‐injected rats compared with controls. We also found that pS404 tau immunoreactivity was higher in 6‐OHDA rats compared with controls. The CA1 region of 6‐OHDA‐injected rats showed increased levels of pS404 tau immunoreactivity, predominantly in the somatodendritic region, which is reminiscent of tau changes during early tauopathy development [78].

The spreading/accumulation of pathological proteins is generally believed to be the first step in the neurodegenerative cascade. Exogenous aSyn PFFs can induce spread of aSyn over time but was not sufficient to induce phosphorylation of endogenous tau along the MFB pathway, at least during the timeframes examined here. This is important because these results may suggest that spread of aSyn alone would result in slow conversion of PD into PDD. In contrast, the Parkinsonian mimetic 6‐OHDA induced phosphorylation of both aSyn and some epitopes of tau in the frontal cortex and the hippocampus within 3 weeks. Although many reports show the oxidative stress‐inducing properties of 6‐OHDA [79, 80], the free radical properties of 6‐OHDA are short‐lived and are unlikely to fully explain the neurodegenerative events observed in the cortex and hippocampus. Indeed, there are increased levels not only of oxidative stress but also nitrative stress in the frontal cortex and the hippocampus.

Synaptic degeneration/neuronal loss is believed to result from the accumulation of pathological proteins as they spread along anatomical connections. We observed a specific loss of the post‐synaptic protein PSD‐95, but not of pre‐synaptic proteins, in the frontal cortex after 6‐OHDA injections, in addition to acute loss of SN neurons within 3 weeks. We do not believe that these effects are related to changes in phosphorylated tau because we observed significant increases in tau phosphorylation at Ser404 in hippocampal sub‐fields but not in the frontal cortex. In contrast, we detected reduced PSD‐95 amounts in the frontal cortex but not the hippocampus. However, we cannot rule out that tau‐directed effects on synapses could be mediated by alterations in tau phosphorylation at other residues or other tau modifications. In addition, perhaps there are other functional changes in tau that were not examined here such as increased tau localisation at synapses. Some neurons along the MFB project to the frontal cortex, and our findings suggest that dying neurons in the SN release toxic substances, including oxidative/nitrative stress mediators that can affect post‐synaptic densities in the frontal cortex. Pre‐synaptic proteins were not affected, and it may be that these are more resilient to oxidative and nitrative stress under these conditions. For aSyn PFFs, we could not detect any loss of synaptic proteins up to 120 days after injection and neither was any significant oxidative/nitrative stress detected in the frontal cortex. However, there was some loss of SN neurons at 120 d.p.i., but not at earlier time points. Thus, oxidative/nitrative stress induced by substantial and rapid neuron loss may potentiate the spread and accumulation of pathological proteins within neural circuits, and therefore, we suggest that this could accelerate the conversion of PD to PDD.

This assertion is further supported by our behavioural tests in 6‐OHDA rats. As a result of TH neuron loss, motor impairment was observed in the asymmetric cylinder test, in line with previous literature [65, 81, 82, 83]. In addition to motor deficits, we found impairments in spatial learning and reference memory in the 6‐OHDA‐injected group, in agreement with a previous report [84].

Taken together, our results demonstrate that (a) neurodegeneration resulting from pathological protein spread may be a slow process which resembles neurodegeneration in human patients; (b) although this process usually starts with spread/accumulation of pathological proteins, the onset of neurodegenerative processes in widespread brain regions can be accelerated by neuronal loss, including upon (c) increased levels of oxidative/nitrative stress related to dying neurons, whereas (d) a gradual process of neuronal loss, such as that resulting from protein aggregate spread, induced minimal oxidative/nitrative stress and resulted in a slow progression of neurodegeneration. We therefore propose that future therapeutic interventions to slow or prevent the conversion of PD to PDD may be through the reduction of oxidative/nitrative stress.

## CONFLICT OF INTEREST

The authors declare no competing interests.

## ETHICAL STATEMENT

All animal studies were approved by HKU and performed in accordance with the Committee on the use of Live animals in teaching and research (CULATR regulations), in which the guidelines of the National Centre for the Replacement, Refinement and Reduction of Animals in Research, UK, and NIH Guidelines for Survival Rodent Surgery, USA are followed. Post‐mortem brain tissue was obtained from the London Neurodegenerative Diseases Brain Bank at King's College London (REC approval 18/WA/0206).

## AUTHOR CONTRIBUTIONS

CCCP performed the experiments and analysed the results. MHS performed the additional 6‐OHDA experiments and behavioural tests. KCL, JQT and VMYL synthesised and provided the PFFs and 81A antibody. They also gave intellectual advice on this study. RCCC and WN designed the study, participated in discussion and supervised the whole study. CCCP, WN and RCCC wrote and revised the manuscript. All authors read and approved the final manuscript.

## Supporting information


**Figure S1:** Illustration depicting the location of the MFB and the key regions of interest. Direct and indirect innervations between anatomical regions are shown. Blue tracts indicate direct projections from the MFB and red tracts indicate indirect inputs to regions of interest. Frontal cortex (FC), striatum (ST), hippocampus (HIPPO) and substantia nigra (SN).

**Figure S2:** **A‐E)** Quantification of the number of phosphorylated α‐synuclein‐immunolabelled cells in both contralateral and ipsilateral hemispheres of different brain regions; frontal cortex (Fc), striatum (St), hippocampus (Hippo), cortex (Crtx) and substantia nigra (SN) at 60, 90 and 120 d.p.i with aSyn PFFs. Unpaired t‐tests were used to determine differences in the number of immunoreactive cells between control and PFF groups for either the contralateral or ipsilateral hemisphere and did not indicate any significant effect at any time points in any of the regions. However, there was a trend where the most abundant phosphorylated aSyn appeared at 90 d.p.i. Data shown is mean ± SEM, n = 3.

**Figure S3:** A) aSyn PFFs did not induce changes in aSyn solubility. Contralateral (indicted by red lines) and ipsilateral (indicated by blue lines) hemispheres of control‐ and aSyn PFF injected rats at 90 d.p.i. were immunoblotted with an antibody against aSyn. C) Charts show the distribution of aSyn in HS, Tx and SS fractions as a proportion of total aSyn.

**Figure S4:** **A)** Immunolabelling of tau phosphorylated at Ser404 (pTau404). Sections were counterstained with haematoxylin in 7 μm paraffin embedded sections of the frontal cortex (Fc), hippocampal subfields CA1 and CA3. aSyn PFFs did not cause an increase in pTau404 immunoreactivity compared to control rats. Images shown are from control and PFF‐injected rats, contralateral (contra) and ipsilateral (ipsi) hemisphere. Scale bars are 20 μm. **B**) Immunolabelling of PHF1 (pTau396/pTau404) and counterstain with haematoxylin in 7 μm paraffin embedded sections of the frontal cortex (Fc), hippocampal subfields CA1 and CA3. aSyn PFFs did not cause a significant difference in PHF1 immunoreactivity compared to controls. Images shown are from control and PFF‐injected rats, contralateral (contra) and ipsilateral (ipsi) hemispheres. Scale bars are 20 μm, n = 1.

**Figure S5:** **A)** aSyn PFFs did not induce changes in tau solubility. Contralateral (indicted by red lines) and ipsilateral (indicated by blue lines) hemispheres of control‐ and aSyn PFF injected rats at 90 d.p.i. were immunoblotted with an antibody against total tau. **C)** Charts show the distribution of tau in HS, Tx and SS fractions as a proportion of total tau.

**Figure S6:** **A, B)** Immunoblots conducted on high salt fractions of contralateral (contra) and ipsilateral (ipsi) frontal cortex and hippocampus of control and aSyn PFF treated rats at 90 d.p.i were probed for post‐ and pre‐synaptic proteins. Blots are representative of frontal cortex (n = 5) and hippocampal (n = 4–6) samples from control and aSyn PFF treated rats, n = 5 for all. No changes in pre‐ or post‐ synaptic markers within the frontal cortex or hippocampal region were found for **C, D)** NR2B, **E,F)** PSD‐95, **G, H)** Synaptotagmin or **I, J)** Synaptophysin. Unpaired t‐tests were used to determine differences in the levels of synaptic proteins between control and PFF groups for either the contralateral or ipsilateral hemisphere and showed no effect of treatment. Data is mean +/− SEM presented as % average control where control is the ipsilateral hemisphere of control‐injected rats.

**Figure S7:** **A)** Graph of phosphorylated aSyn load in various brain regions after 6−ΟΗDA injection into the MFB. No cells immunoreactive for phosphorylated aSyn were found in control injected tissues. Frontal cortex (Fc), striatum (ST), hippocampus (Hippo), cortex (Crtx) and substantia nigra (SN). Data is mean ± SEM, n = 3.

**Figure S8:** Representative images of sections from control and 6‐OHDA‐injected rats that were collected 1‐week after injection following immunolabelling with an antibody against aSyn phosphorylated at Ser129. Sections were counterstained with haematoxylin. No phosphorylated aSyn was detected in either hemisphere of the frontal cortex (Fc), CA1 and CA3 regions of the hippocampus, or in the substantia nigra (SN). Scale bars = 20 μm, n = 3.

**Figure S9:** **A)** PHF1 immunolabelling of formaldehyde fixed, paraffin embedded 7 μm sections of postmortem human control and AD brain. Inset shows higher magnification of immunoreactive neurons. **B)** Control animals showed limited positivity for PHF1, while **C)** 6‐OHDA injected animals showed positive PHF1 staining in both contralateral (contra) and ipsilateral (ipsi) sides. Representative images are shown. Purple scale bar: 5 μm; black scale bar: 500 μm. **D)** Quantification of the number of PHF1 positive cells per mm2 in the CA1 and **E)** CA3. **F)** PHF1 immunolabelling of frontal cortex. **G)** Quantification of PHF1 immunoreactivity in the frontal cortex. Unpaired t‐tests were used to determine differences in the levels of immunoreactivity between control and 6‐OHDA groups for either the contralateral or ipsilateral hemisphere and showed no effect of treatment. Data is mean ± SEM, n = 3​.

**Figure S10:** **A‐B)** Representative immunoblots of samples from contralateral (contra) and ipsilateral (ipsi) hemispheres from control and 6‐OHDA injected rats three weeks after injection. Blots were probed with antibodies against NR2B (150 kDa), PSD‐95 (95 kDa), synaptophysin (38 kDa) and synaptotagmin (60 kDa), with β‐actin (42 kDa) used as a loading control. Graphs show quantification of frontal cortex synaptic proteins relative to β‐actin for **C)** NR2B (control, n = 5, 6‐OHDA, n = 5), **E)** Synaptotagmin (control, n = 5, PFF, n = 4) and **G)** Synaptophysin (control, n = 6, 6‐OHDA, n = 4). Graphs show quantification of hippocampal synaptic proteins relative to β‐actin for **D)** NR2B (control, n = 6, 6‐OHDA, n = 4). **F)** Synaptotagmin (n = 6) and **H)** Synaptophysin (control, n = 5, 6‐OHDA, n = 6). Unpaired t‐tests were used to determine differences in the levels of synaptic proteins between control and 6‐OHDA groups for either the contralateral or ipsilateral hemisphere and showed no effect of treatment. Data is mean +/− SEM presented as % average control where control is the ipsilateral hemisphere of control‐injected rats.

**Figure S11:** **A)** Immunolabelling of 8‐hydroxyguanosine (8OHG) to detect oxidative DNA/RNA damage in 7 μm paraffin embedded sections of the frontal cortex (Fc), hippocampal subfields CA1 and CA3 from aSyn PFF and control injected rats 90 d.p.i. Black arrows indicate labelled cells. Quantification of the percentage area of positive 8OHG cells within the **B)** frontal cortex, **C)** CA1 and **D)** CA3 of control and aSyn PFF injected rats. Unpaired t‐tests were used to determine differences in the levels of immunoreactivity between control and PFF groups for either the contralateral or ipsilateral hemisphere and showed no effect of treatment. Data is mean ± SEM, n = 3. **D)** Immunolabelling of nitrotyrosine positive cells to detect for cell damage and inflammation, on 7 μm paraffin embedded sections from the frontal and hippocampal subfield CA1 and CA3 regions. aSyn PFF injected rats did not show any positive nitrotyrosine cells in the frontal cortex or hippocampal subfields (CA1) and (CA3), in either ipsi or contra hemisphere. Control rats not shown. Scale bar 20 μm.

**Figure S12:** A) Rat brain sections (7 μm) were immunolabelled with an antibody against TH to detect dopaminergic neurons in sections of SN from control and 6‐OHDA treated rats, 1‐week post‐injection with 6‐OHDA. Images were obtained using Akoya Vectra Polaris Imaging System; representative images were chosen for each experimental group. B) No significant differences were observed in the number of dopaminergic neurons in SN between control and 6‐OHDA groups, or between contralateral and ipsilateral hemispheres (n = 3). Data is mean +/− SEM.

**Figure S13:** **A)** Immunolabelling of 8‐hydroxyguanosine (8OHG) to detect oxidative DNA/RNA damage in 7 μm paraffin embedded sections of the frontal cortex (Fc), hippocampal subfields CA1 and CA3 from 6‐OHDA and control injected rats 1 week post injection. Scale bar is 20 μm. Quantification of the percentage area of positive 8OHG cells within the **B)** frontal cortex, **C)** CA1, **D)** CA3 and **E)** SN of control and 6‐OHDA injected rats. Unpaired t‐tests were used to determine differences in the levels of immunoreactivity between control and PFF groups for either the contralateral or ipsilateral hemisphere and showed no effect of treatment for the cortex, CA1 or CA3, and a significant increase in 8OHG in the SN of 6‐OHDA injected rats. **P < 0.01. n = 3 (control), n = 4 (6‐OHDA).

**Figure S14:** **A)** Transient asymmetric forelimb deficits in aSyn PFF injected rats. An asymmetric cylinder test was used to determine if there are changes in contralateral forelimb usage following injection of rat MFB with α‐synuclein PFFs. Rats were tested post‐surgery at 60, 90, and 120 d.p.i. Bar charts show percentage of contralateral forepaw usage at 90 d.p.i. (control n = 7, PFF n = 8). Data are mean ± SEM, * p < 0.05. Statistical analysis used was unpaired t‐test. **B)** Rats injected with aSyn PFFs show no impairment in their learning ability on the MWM task compared to control rats as shown by escape latency on each of the five days of training. **C)** The number of times that rats cross the target area from which the platform was removed was determined as a measure of visuospatial memory during the probe test. **D)** graph shows the time spent in each quadrant during the probe trial at 90 d.p.i. (n = 7). Analysis for **(B, D)** repeated 2‐way ANOVA, unpaired t‐test **(A,C).** Data shown is mean ± SEM.

**Figure S15:** **A)** Measures of distance swum during the Morris water maze task between control and 6‐OHDA injected animals. 6‐OHDA injected animals exhibited a reduced distance swum during the task compared to control injected animals. Data is mean ± SEM, statistical analysis used was two‐tailed unpaired t‐test (control n = 10, 6‐OHDA, n = 10).

**Figure S16:** **A‐B)** Measures of time spent in seconds inside the centre zone as an indication of anxiety‐like behaviour using the open field test shows no difference between control and 6‐OHDA groups or α‐synuclein PFF and control groups. **C‐D)** Land‐based locomotor activity was measured as total distance travelled in centimetres. Control and 6‐OHDA rats and α‐synuclein PFF and control rats travelled similar distances. Graphs show trajectories of representative **E)** Control and aSyn PFF, and **F)** Control and 6‐OHDA‐treated rats during the open field task. The yellow box represents the outer zone, the blue square is the middle zone and the red box indicates the inner zone of the open field arena. Data is mean ± SEM, statistical analysis used was two‐tailed unpaired t‐test (control n = 6, 6‐OHDA, n = 4​, control for PFFs n = 8, PFFs, n = 8).

## Data Availability

The data that support the findings of this study are available from the corresponding author upon reasonable request.
