# Supplementary figures and images for: Investigating key factors underlying neurodegeneration linked to alpha‐synuclein spread
Source: Neuropathol Appl Neurobiol. 2022 Jul 4;48(6):e12829. doi: 10.1111/nan.12829 (PMC9546483; doi:10.1111/nan.12829)

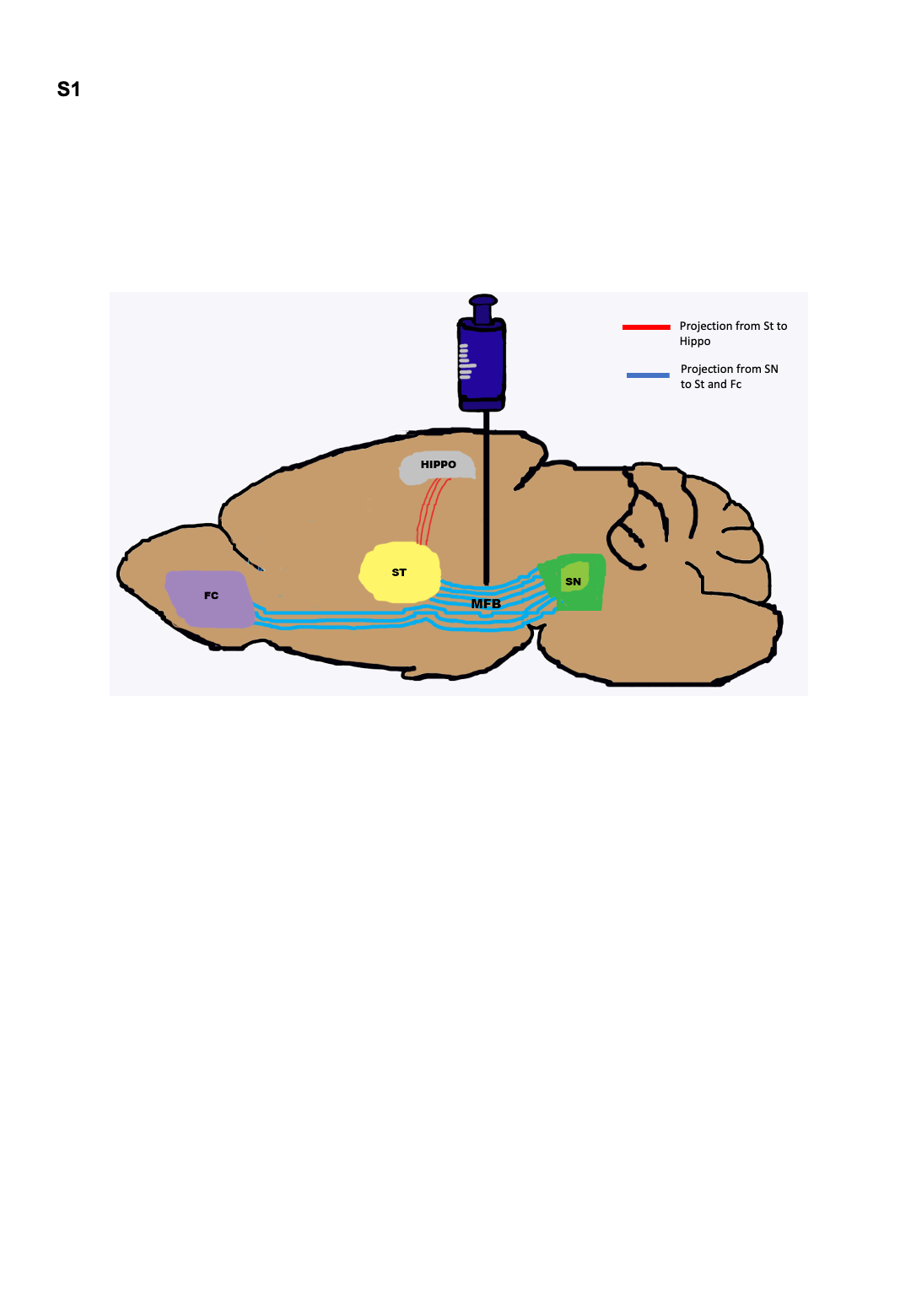

Supplement: Supplementary file 1 — Figure S1: Illustration depicting the location of the MFB and the key regions of interest. Direct and indirect innervations between anatomical regions are shown. Blue tracts indicate direct projections from the MFB and red tracts indicate indirect inputs to regions of interest. Frontal cortex (FC), striatum (ST), hippocampus (HIPPO) and substantia nigra (SN). [file NAN-48-0-s016.tiff]

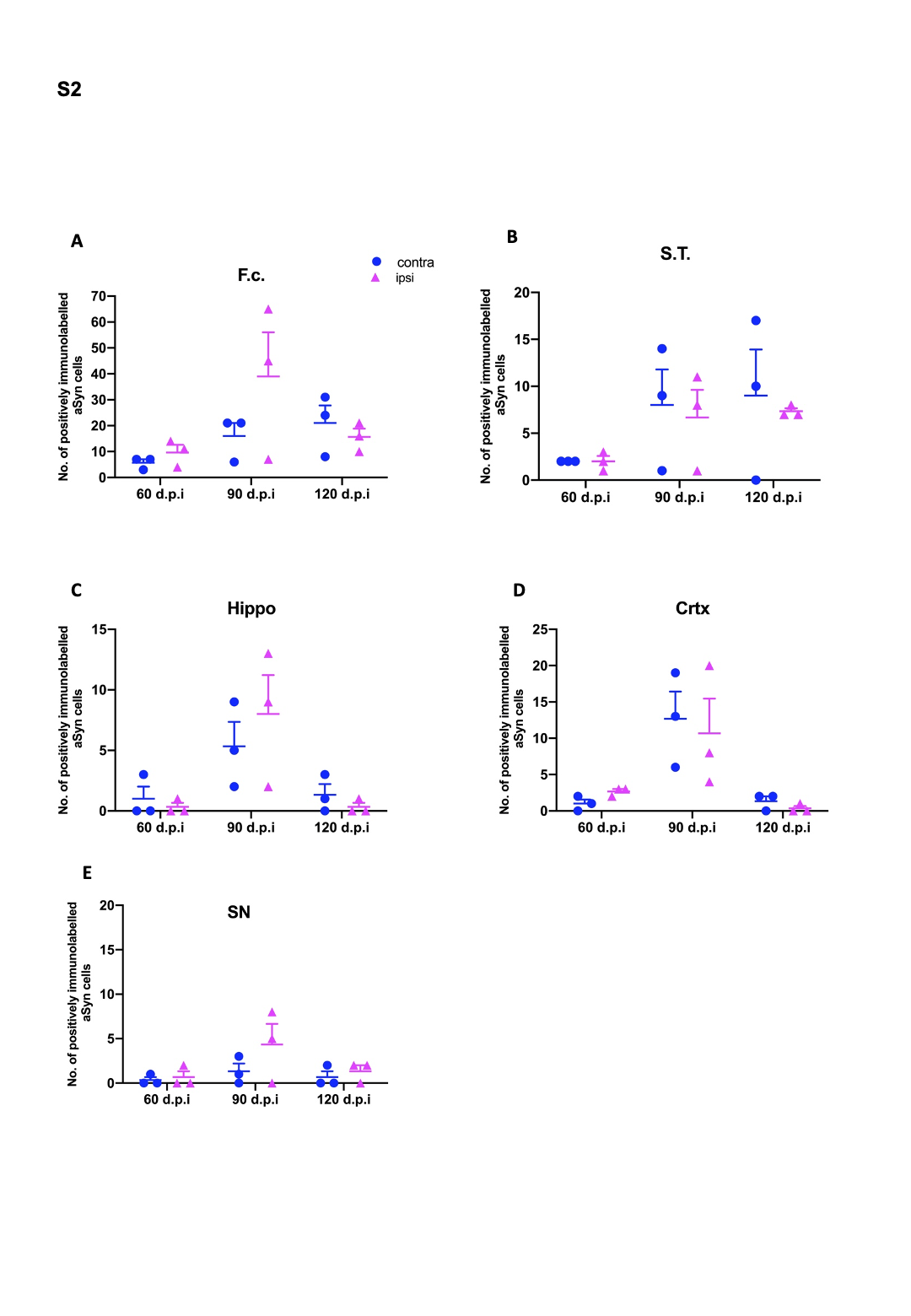

Supplement: Supplementary file 2 — Figure S2: A‐E) Quantification of the number of phosphorylated α‐synuclein‐immunolabelled cells in both contralateral and ipsilateral hemispheres of different brain regions; frontal cortex (Fc), striatum (St), hippocampus (Hippo), cortex (Crtx) and substantia nigra (SN) at 60, 90 and 120 d.p.i with aSyn PFFs. Unpaired t‐tests were used to determine differences in the number of immunoreactive cells between control and PFF groups for either the contralateral or ipsilateral hemisphere and did not indicate any significant effect at any time points in any of the regions. However, there was a trend where the most abundant phosphorylated aSyn appeared at 90 d.p.i. Data shown is mean ± SEM, n = 3. [file NAN-48-0-s015.tiff]

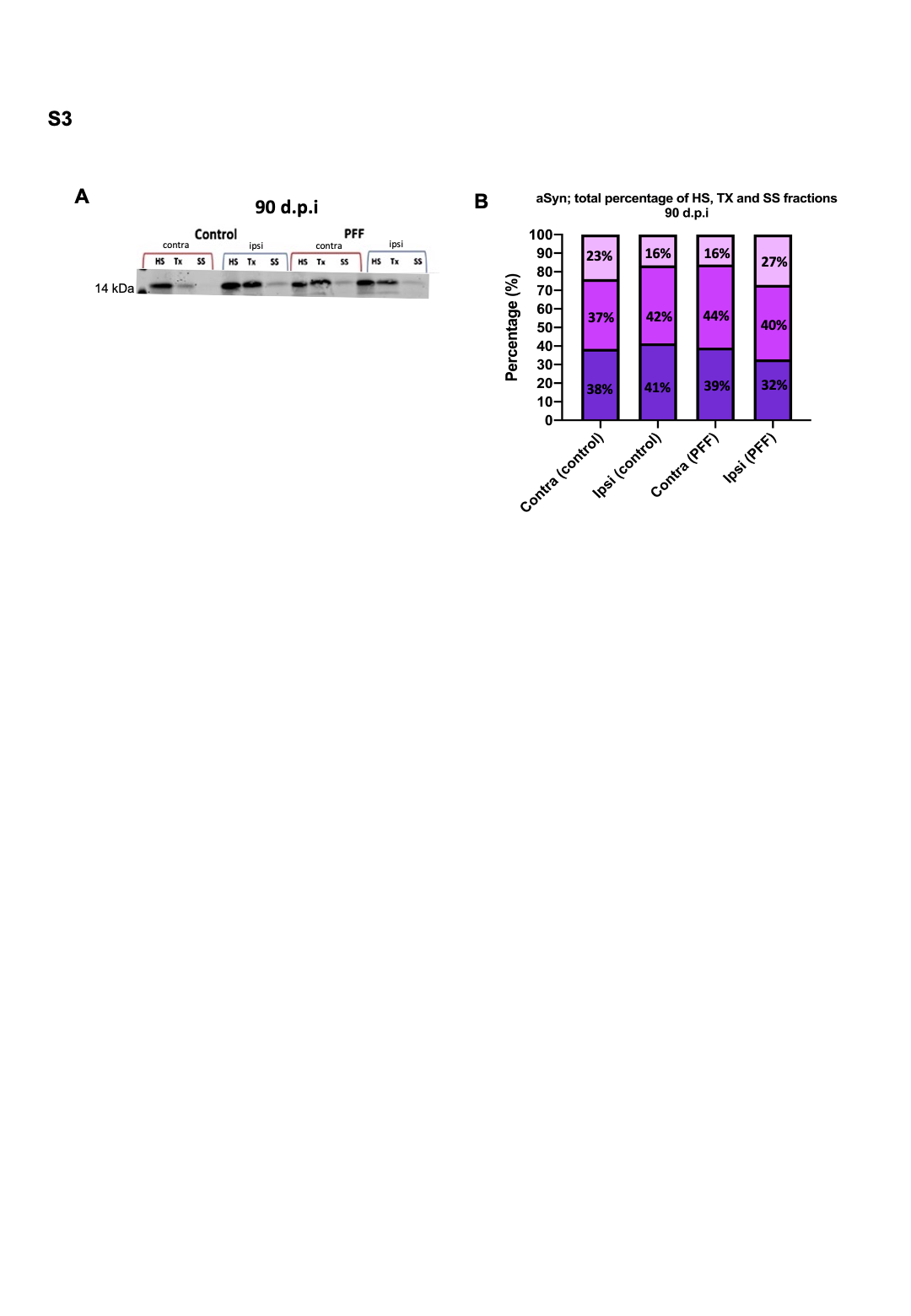

Supplement: Supplementary file 3 — Figure S3: A) aSyn PFFs did not induce changes in aSyn solubility. Contralateral (indicted by red lines) and ipsilateral (indicated by blue lines) hemispheres of control‐ and aSyn PFF injected rats at 90 d.p.i. were immunoblotted with an antibody against aSyn. C) Charts show the distribution of aSyn in HS, Tx and SS fractions as a proportion of total aSyn. [file NAN-48-0-s013.tiff]

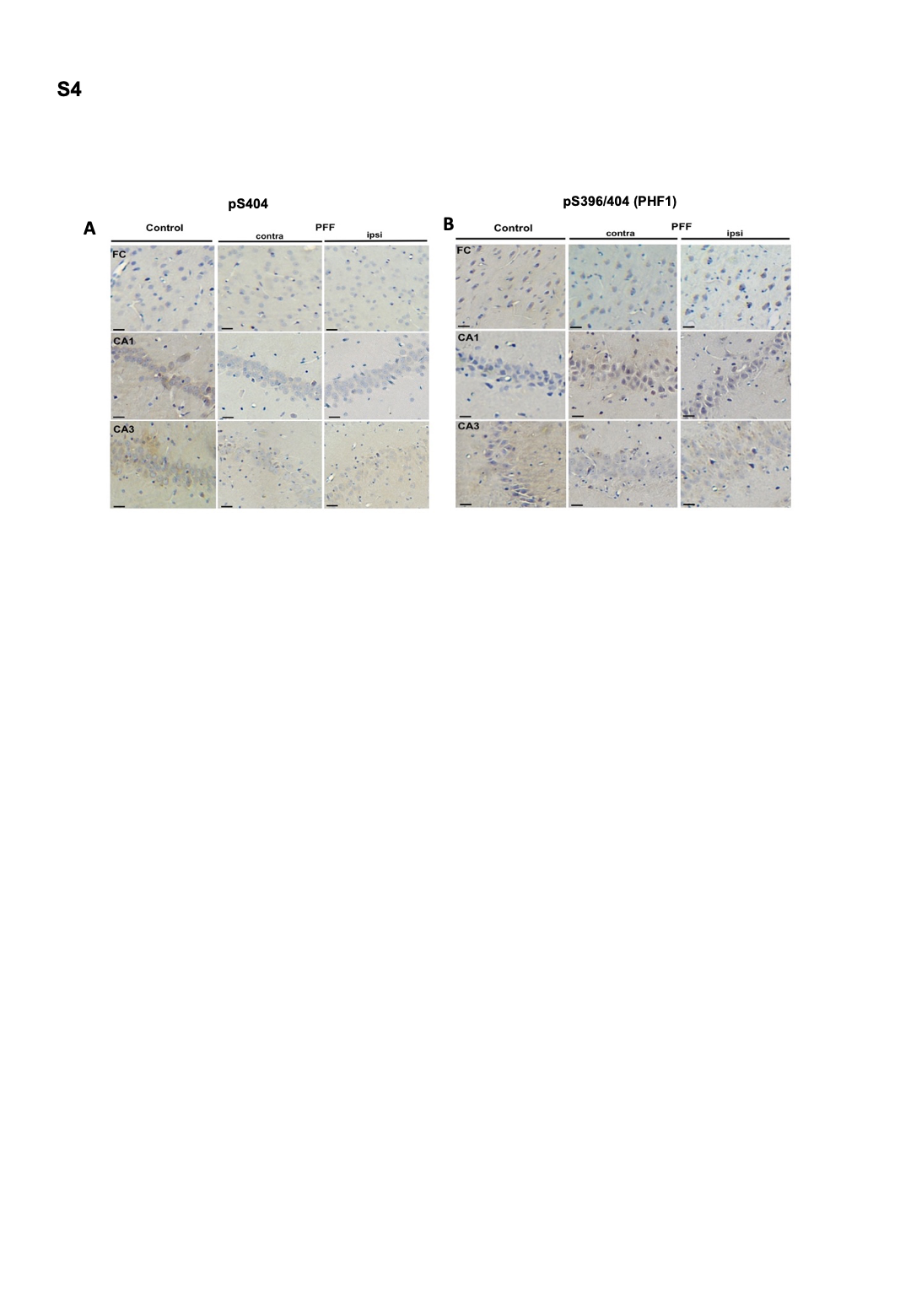

Supplement: Supplementary file 4 — Figure S4: A) Immunolabelling of tau phosphorylated at Ser404 (pTau404). Sections were counterstained with haematoxylin in 7 μm paraffin embedded sections of the frontal cortex (Fc), hippocampal subfields CA1 and CA3. aSyn PFFs did not cause an increase in pTau404 immunoreactivity compared to control rats. Images shown are from control and PFF‐injected rats, contralateral (contra) and ipsilateral (ipsi) hemisphere. Scale bars are 20 μm. B) Immunolabelling of PHF1 (pTau396/pTau404) and counterstain with haematoxylin in 7 μm paraffin embedded sections of the frontal cortex (Fc), hippocampal subfields CA1 and CA3. aSyn PFFs did not cause a significant difference in PHF1 immunoreactivity compared to controls. Images shown are from control and PFF‐injected rats, contralateral (contra) and ipsilateral (ipsi) hemispheres. Scale bars are 20 μm, n = 1. [file NAN-48-0-s002.tiff]

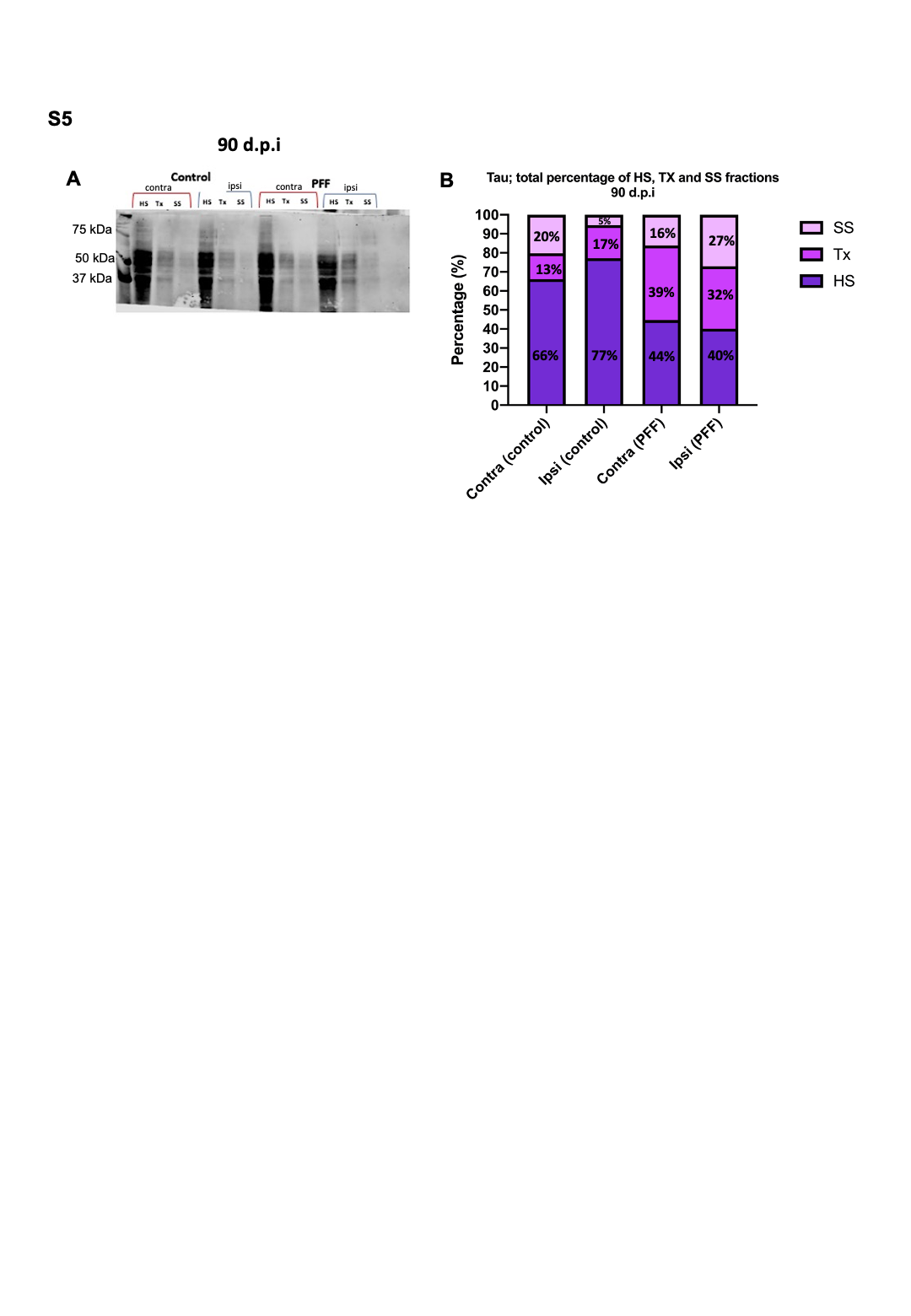

Supplement: Supplementary file 5 — Figure S5: A) aSyn PFFs did not induce changes in tau solubility. Contralateral (indicted by red lines) and ipsilateral (indicated by blue lines) hemispheres of control‐ and aSyn PFF injected rats at 90 d.p.i. were immunoblotted with an antibody against total tau. C) Charts show the distribution of tau in HS, Tx and SS fractions as a proportion of total tau. [file NAN-48-0-s003.tiff]

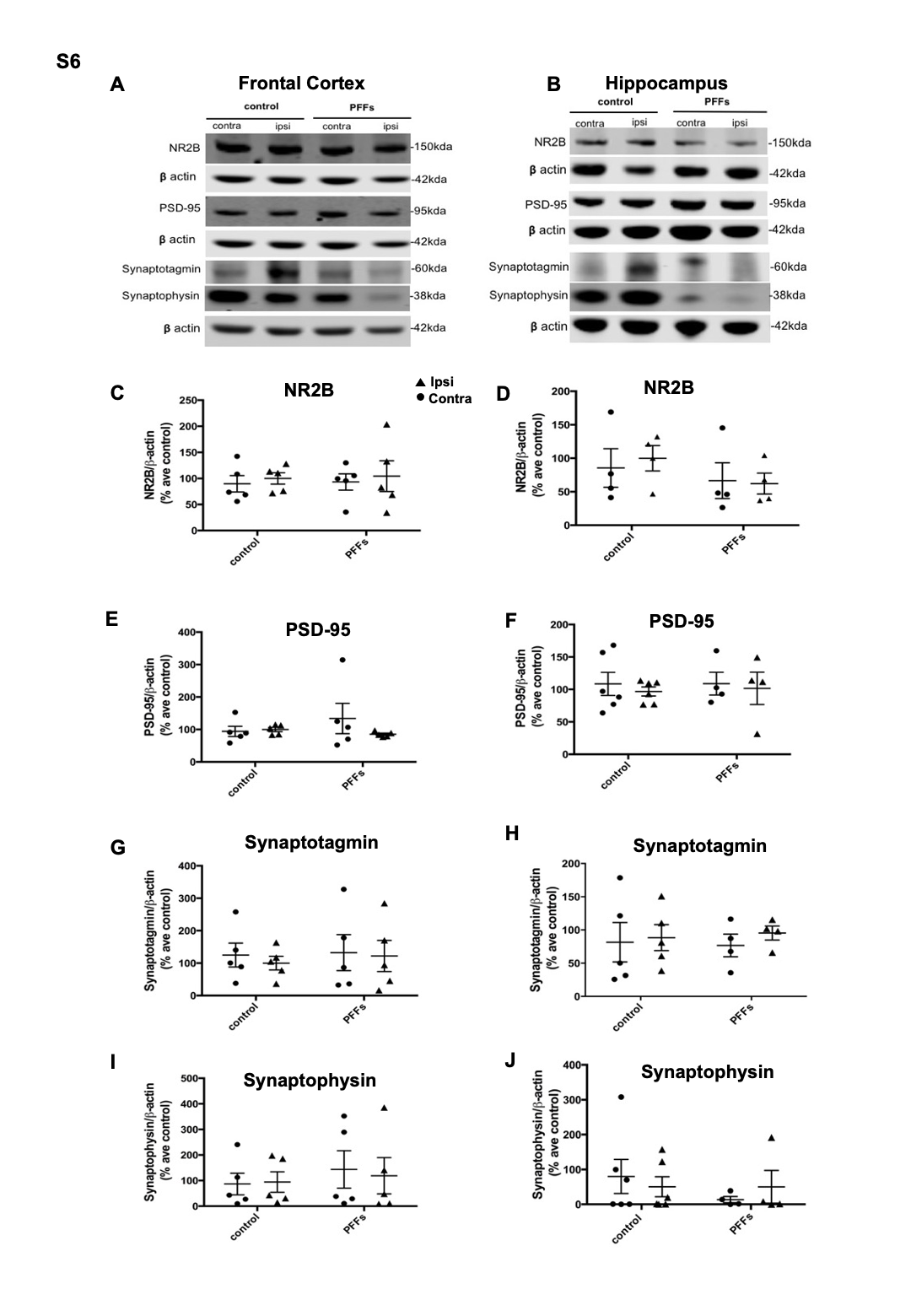

Supplement: Supplementary file 6 — Figure S6: A, B) Immunoblots conducted on high salt fractions of contralateral (contra) and ipsilateral (ipsi) frontal cortex and hippocampus of control and aSyn PFF treated rats at 90 d.p.i were probed for post‐ and pre‐synaptic proteins. Blots are representative of frontal cortex (n = 5) and hippocampal (n = 4–6) samples from control and aSyn PFF treated rats, n = 5 for all. No changes in pre‐ or post‐ synaptic markers within the frontal cortex or hippocampal region were found for C, D) NR2B, E,F) PSD‐95, G, H) Synaptotagmin or I, J) Synaptophysin. Unpaired t‐tests were used to determine differences in the levels of synaptic proteins between control and PFF groups for either the contralateral or ipsilateral hemisphere and showed no effect of treatment. Data is mean +/− SEM presented as % average control where control is the ipsilateral hemisphere of control‐injected rats. [file NAN-48-0-s009.tiff]

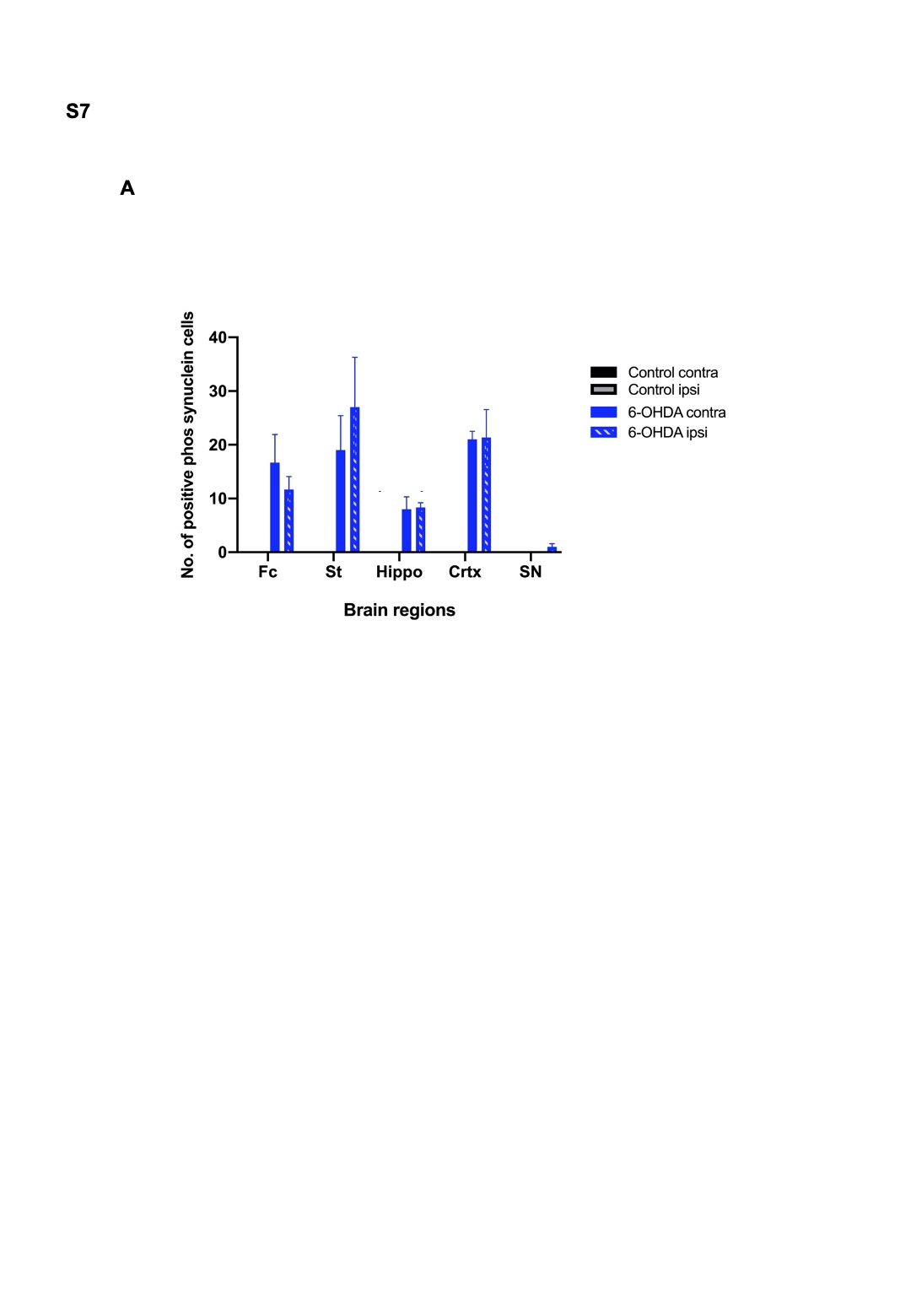

Supplement: Supplementary file 7 — Figure S7: A) Graph of phosphorylated aSyn load in various brain regions after 6−ΟΗDA injection into the MFB. No cells immunoreactive for phosphorylated aSyn were found in control injected tissues. Frontal cortex (Fc), striatum (ST), hippocampus (Hippo), cortex (Crtx) and substantia nigra (SN). Data is mean ± SEM, n = 3. [file NAN-48-0-s006.tiff]

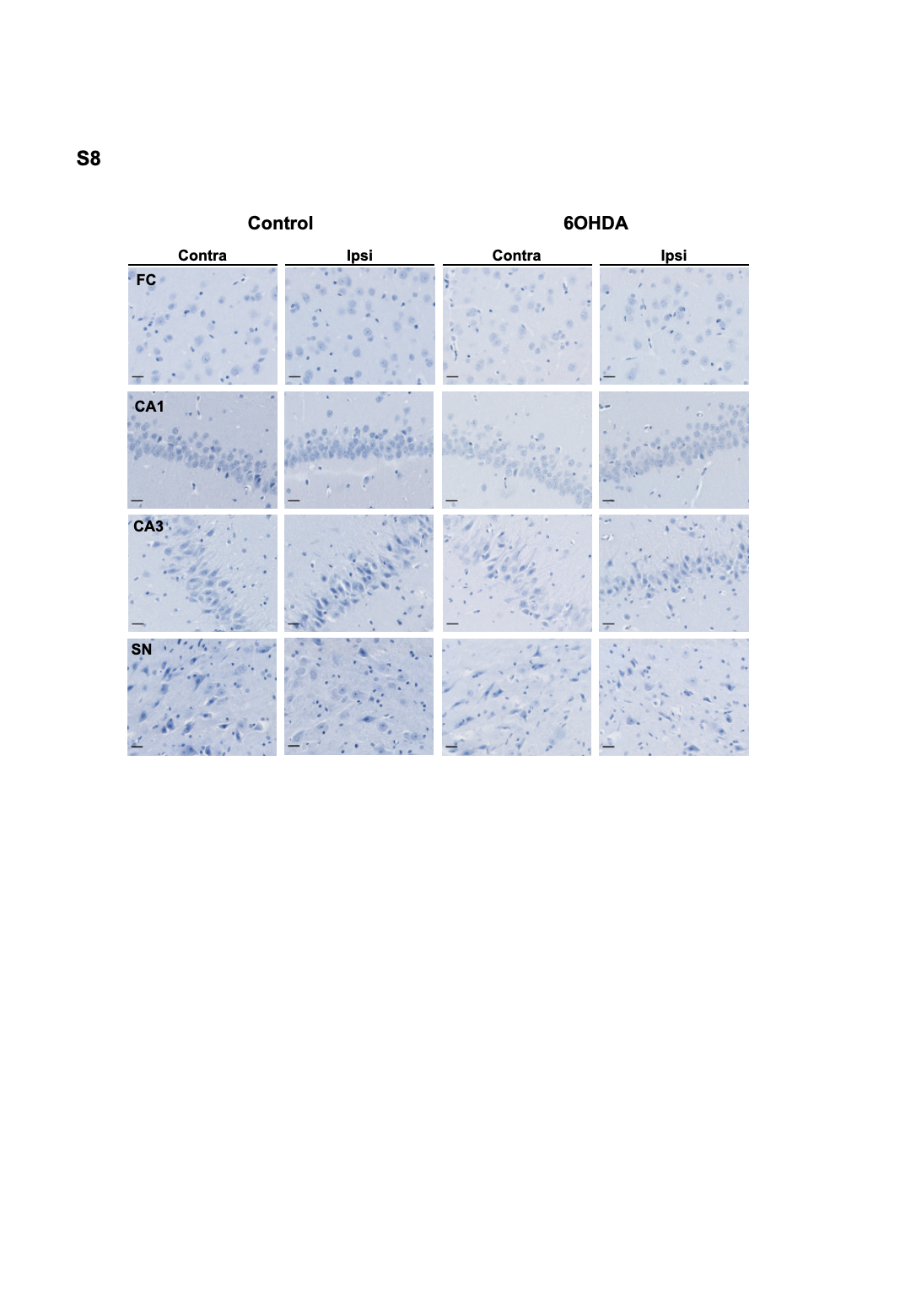

Supplement: Supplementary file 8 — Figure S8: Representative images of sections from control and 6‐OHDA‐injected rats that were collected 1‐week after injection following immunolabelling with an antibody against aSyn phosphorylated at Ser129. Sections were counterstained with haematoxylin. No phosphorylated aSyn was detected in either hemisphere of the frontal cortex (Fc), CA1 and CA3 regions of the hippocampus, or in the substantia nigra (SN). Scale bars = 20 μm, n = 3. [file NAN-48-0-s012.tiff]

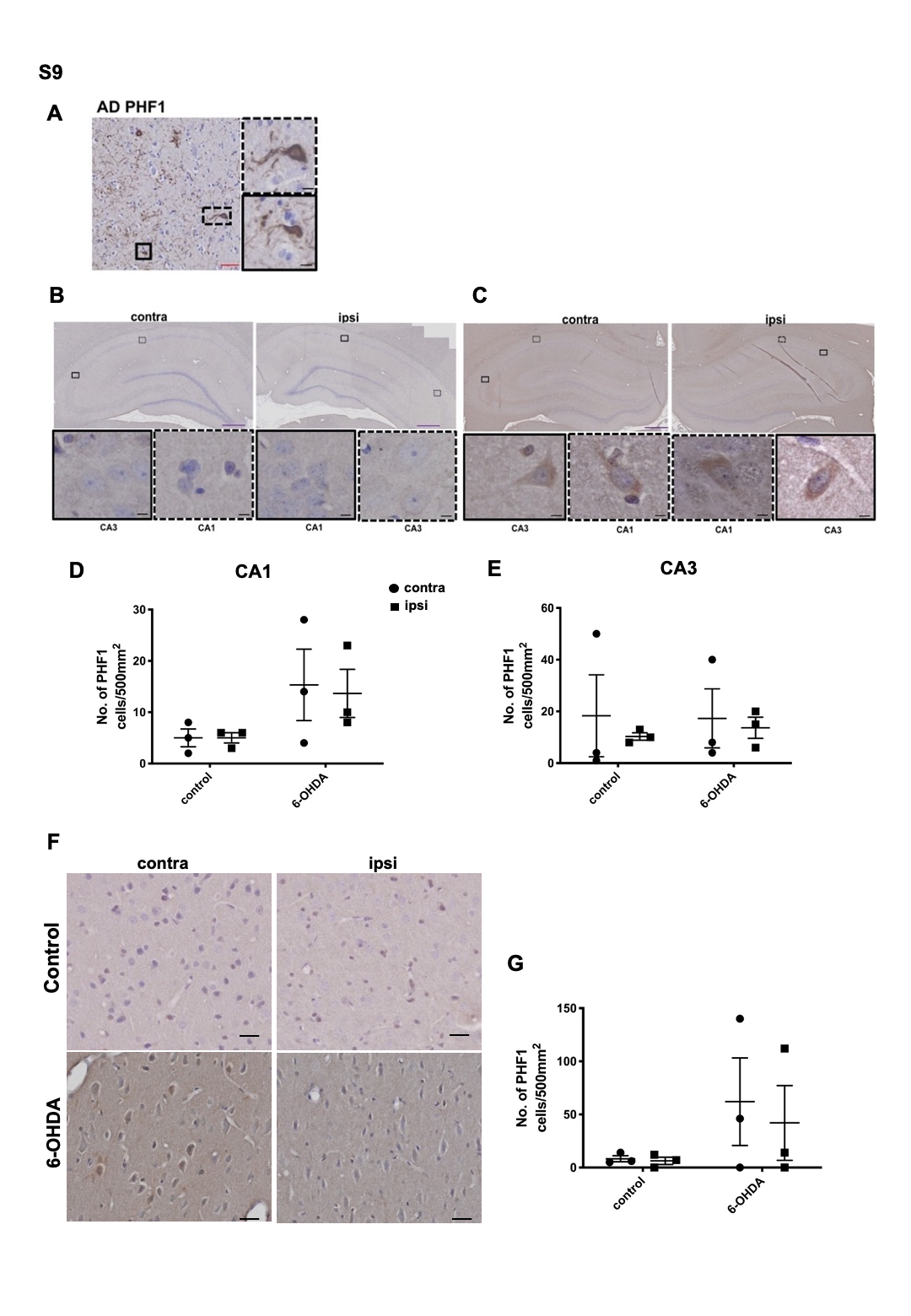

Supplement: Supplementary file 9 — Figure S9: A) PHF1 immunolabelling of formaldehyde fixed, paraffin embedded 7 μm sections of postmortem human control and AD brain. Inset shows higher magnification of immunoreactive neurons. B) Control animals showed limited positivity for PHF1, while C) 6‐OHDA injected animals showed positive PHF1 staining in both contralateral (contra) and ipsilateral (ipsi) sides. Representative images are shown. Purple scale bar: 5 μm; black scale bar: 500 μm. D) Quantification of the number of PHF1 positive cells per mm2 in the CA1 and E) CA3. F) PHF1 immunolabelling of frontal cortex. G) Quantification of PHF1 immunoreactivity in the frontal cortex. Unpaired t‐tests were used to determine differences in the levels of immunoreactivity between control and 6‐OHDA groups for either the contralateral or ipsilateral hemisphere and showed no effect of treatment. Data is mean ± SEM, n = 3​. [file NAN-48-0-s007.tiff]

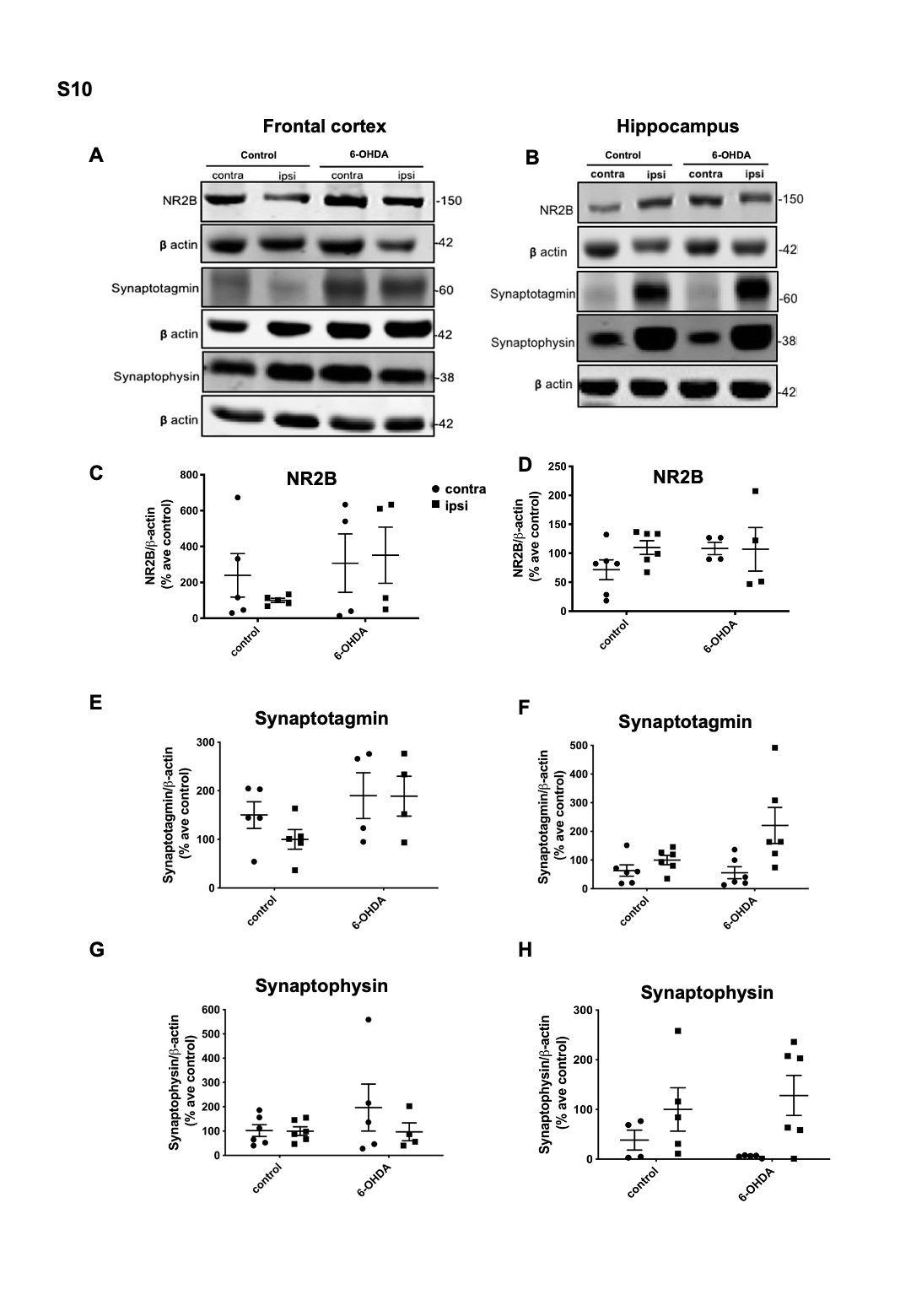

Supplement: Supplementary file 10 — Figure S10: A‐B) Representative immunoblots of samples from contralateral (contra) and ipsilateral (ipsi) hemispheres from control and 6‐OHDA injected rats three weeks after injection. Blots were probed with antibodies against NR2B (150 kDa), PSD‐95 (95 kDa), synaptophysin (38 kDa) and synaptotagmin (60 kDa), with β‐actin (42 kDa) used as a loading control. Graphs show quantification of frontal cortex synaptic proteins relative to β‐actin for C) NR2B (control, n = 5, 6‐OHDA, n = 5), E) Synaptotagmin (control, n = 5, PFF, n = 4) and G) Synaptophysin (control, n = 6, 6‐OHDA, n = 4). Graphs show quantification of hippocampal synaptic proteins relative to β‐actin for D) NR2B (control, n = 6, 6‐OHDA, n = 4). F) Synaptotagmin (n = 6) and H) Synaptophysin (control, n = 5, 6‐OHDA, n = 6). Unpaired t‐tests were used to determine differences in the levels of synaptic proteins between control and 6‐OHDA groups for either the contralateral or ipsilateral hemisphere and showed no effect of treatment. Data is mean +/− SEM presented as % average control where control is the ipsilateral hemisphere of control‐injected rats. [file NAN-48-0-s008.tiff]

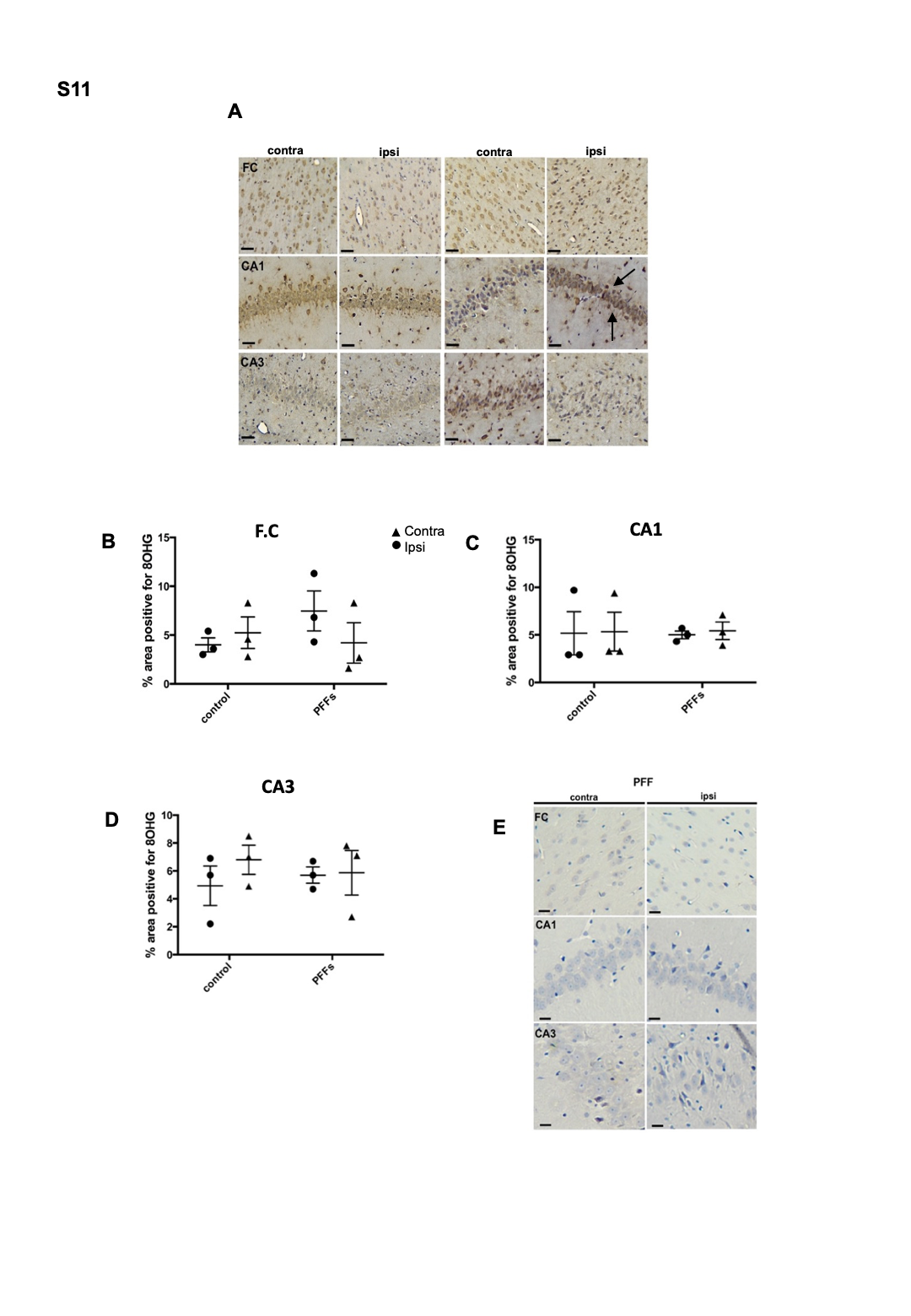

Supplement: Supplementary file 11 — Figure S11: A) Immunolabelling of 8‐hydroxyguanosine (8OHG) to detect oxidative DNA/RNA damage in 7 μm paraffin embedded sections of the frontal cortex (Fc), hippocampal subfields CA1 and CA3 from aSyn PFF and control injected rats 90 d.p.i. Black arrows indicate labelled cells. Quantification of the percentage area of positive 8OHG cells within the B) frontal cortex, C) CA1 and D) CA3 of control and aSyn PFF injected rats. Unpaired t‐tests were used to determine differences in the levels of immunoreactivity between control and PFF groups for either the contralateral or ipsilateral hemisphere and showed no effect of treatment. Data is mean ± SEM, n = 3. D) Immunolabelling of nitrotyrosine positive cells to detect for cell damage and inflammation, on 7 μm paraffin embedded sections from the frontal and hippocampal subfield CA1 and CA3 regions. aSyn PFF injected rats did not show any positive nitrotyrosine cells in the frontal cortex or hippocampal subfields (CA1) and (CA3), in either ipsi or contra hemisphere. Control rats not shown. Scale bar 20 μm. [file NAN-48-0-s010.tiff]

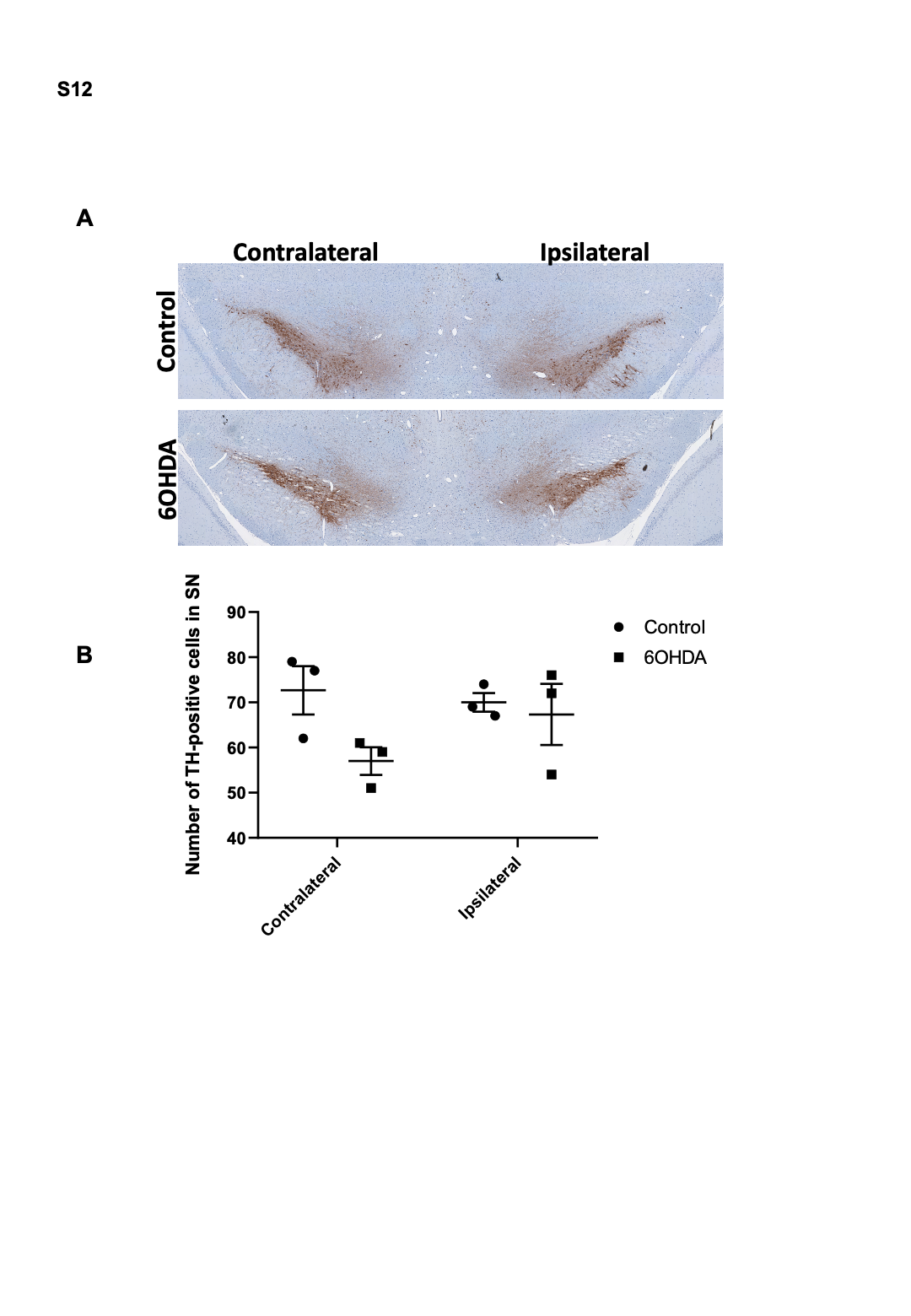

Supplement: Supplementary file 12 — Figure S12: A) Rat brain sections (7 μm) were immunolabelled with an antibody against TH to detect dopaminergic neurons in sections of SN from control and 6‐OHDA treated rats, 1‐week post‐injection with 6‐OHDA. Images were obtained using Akoya Vectra Polaris Imaging System; representative images were chosen for each experimental group. B) No significant differences were observed in the number of dopaminergic neurons in SN between control and 6‐OHDA groups, or between contralateral and ipsilateral hemispheres (n = 3). Data is mean +/− SEM. [file NAN-48-0-s005.tiff]

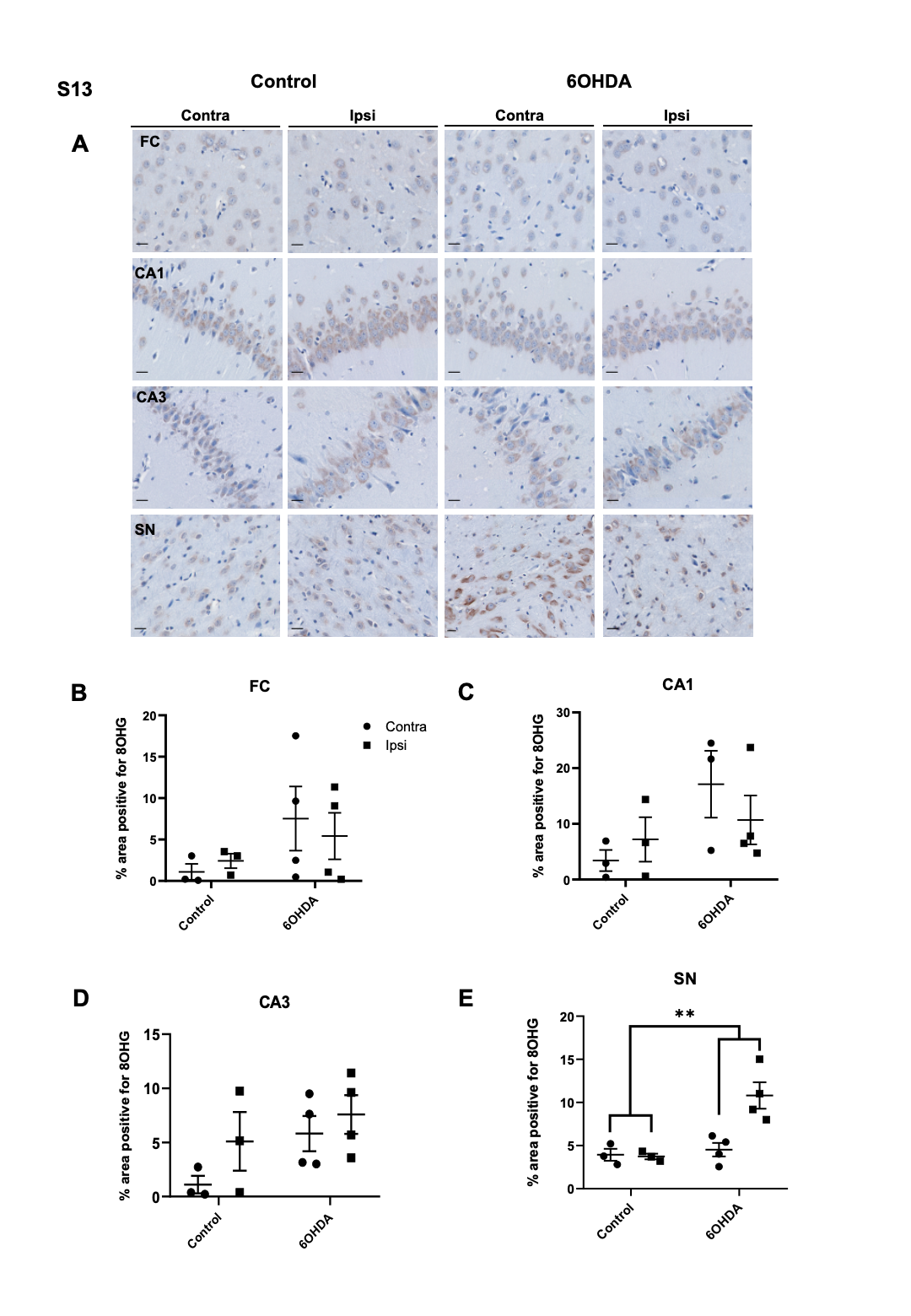

Supplement: Supplementary file 13 — Figure S13: A) Immunolabelling of 8‐hydroxyguanosine (8OHG) to detect oxidative DNA/RNA damage in 7 μm paraffin embedded sections of the frontal cortex (Fc), hippocampal subfields CA1 and CA3 from 6‐OHDA and control injected rats 1 week post injection. Scale bar is 20 μm. Quantification of the percentage area of positive 8OHG cells within the B) frontal cortex, C) CA1, D) CA3 and E) SN of control and 6‐OHDA injected rats. Unpaired t‐tests were used to determine differences in the levels of immunoreactivity between control and PFF groups for either the contralateral or ipsilateral hemisphere and showed no effect of treatment for the cortex, CA1 or CA3, and a significant increase in 8OHG in the SN of 6‐OHDA injected rats. **P < 0.01. n = 3 (control), n = 4 (6‐OHDA). [file NAN-48-0-s014.tiff]

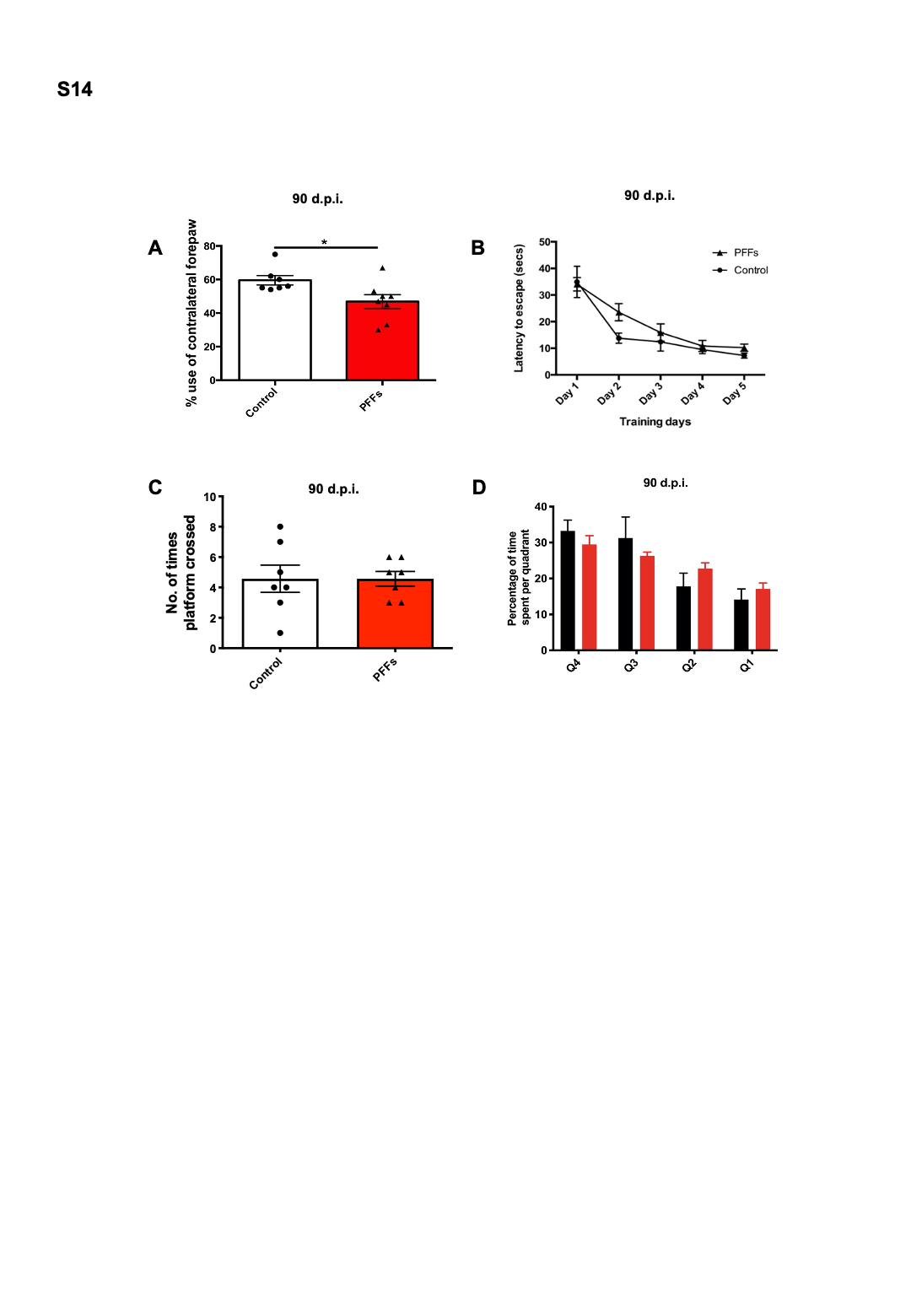

Supplement: Supplementary file 14 — Figure S14: A) Transient asymmetric forelimb deficits in aSyn PFF injected rats. An asymmetric cylinder test was used to determine if there are changes in contralateral forelimb usage following injection of rat MFB with α‐synuclein PFFs. Rats were tested post‐surgery at 60, 90, and 120 d.p.i. Bar charts show percentage of contralateral forepaw usage at 90 d.p.i. (control n = 7, PFF n = 8). Data are mean ± SEM, * p < 0.05. Statistical analysis used was unpaired t‐test. B) Rats injected with aSyn PFFs show no impairment in their learning ability on the MWM task compared to control rats as shown by escape latency on each of the five days of training. C) The number of times that rats cross the target area from which the platform was removed was determined as a measure of visuospatial memory during the probe test. D) graph shows the time spent in each quadrant during the probe trial at 90 d.p.i. (n = 7). Analysis for (B, D) repeated 2‐way ANOVA, unpaired t‐test (A,C). Data shown is mean ± SEM. [file NAN-48-0-s001.tiff]

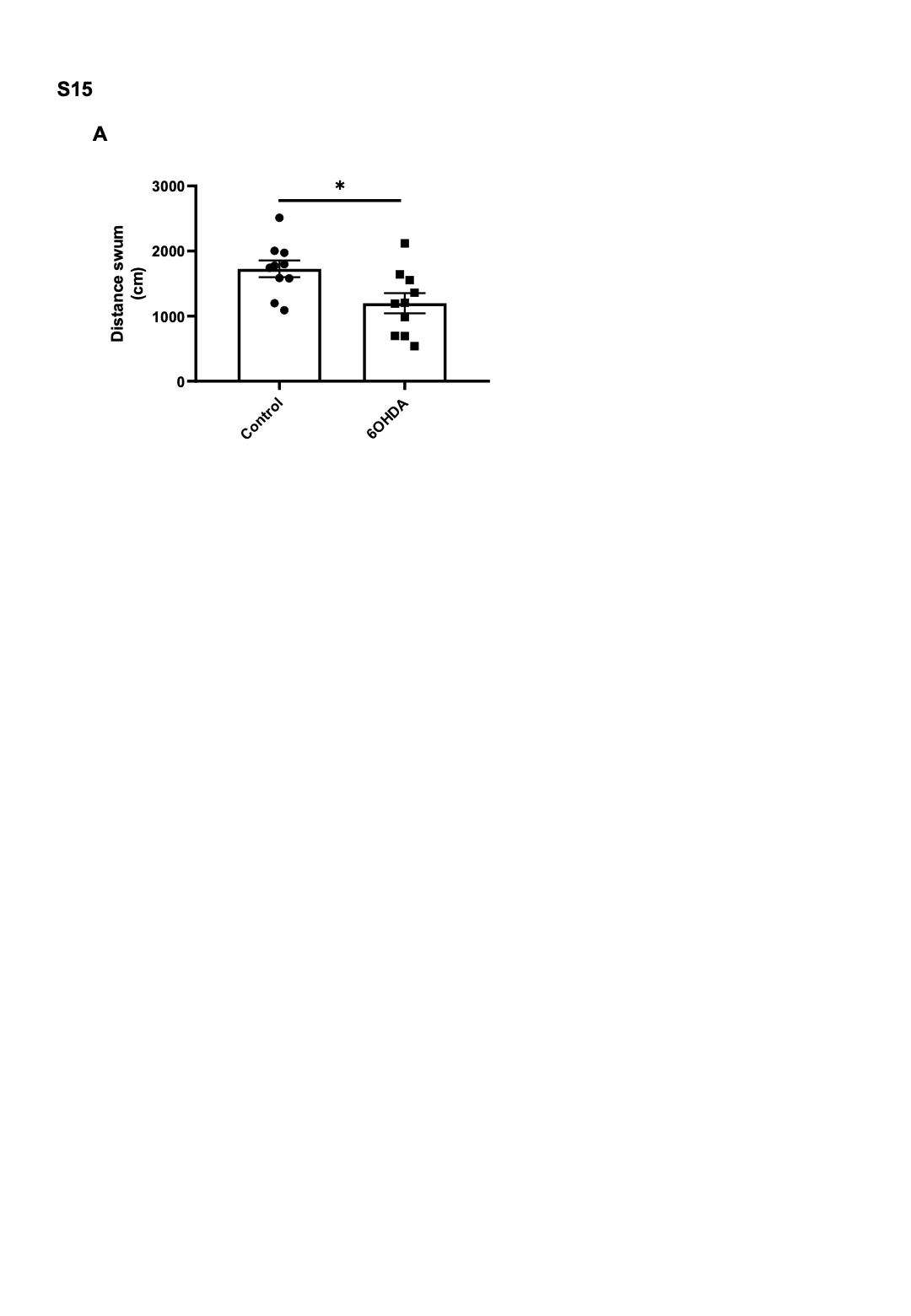

Supplement: Supplementary file 15 — Figure S15: A) Measures of distance swum during the Morris water maze task between control and 6‐OHDA injected animals. 6‐OHDA injected animals exhibited a reduced distance swum during the task compared to control injected animals. Data is mean ± SEM, statistical analysis used was two‐tailed unpaired t‐test (control n = 10, 6‐OHDA, n = 10). [file NAN-48-0-s004.tiff]

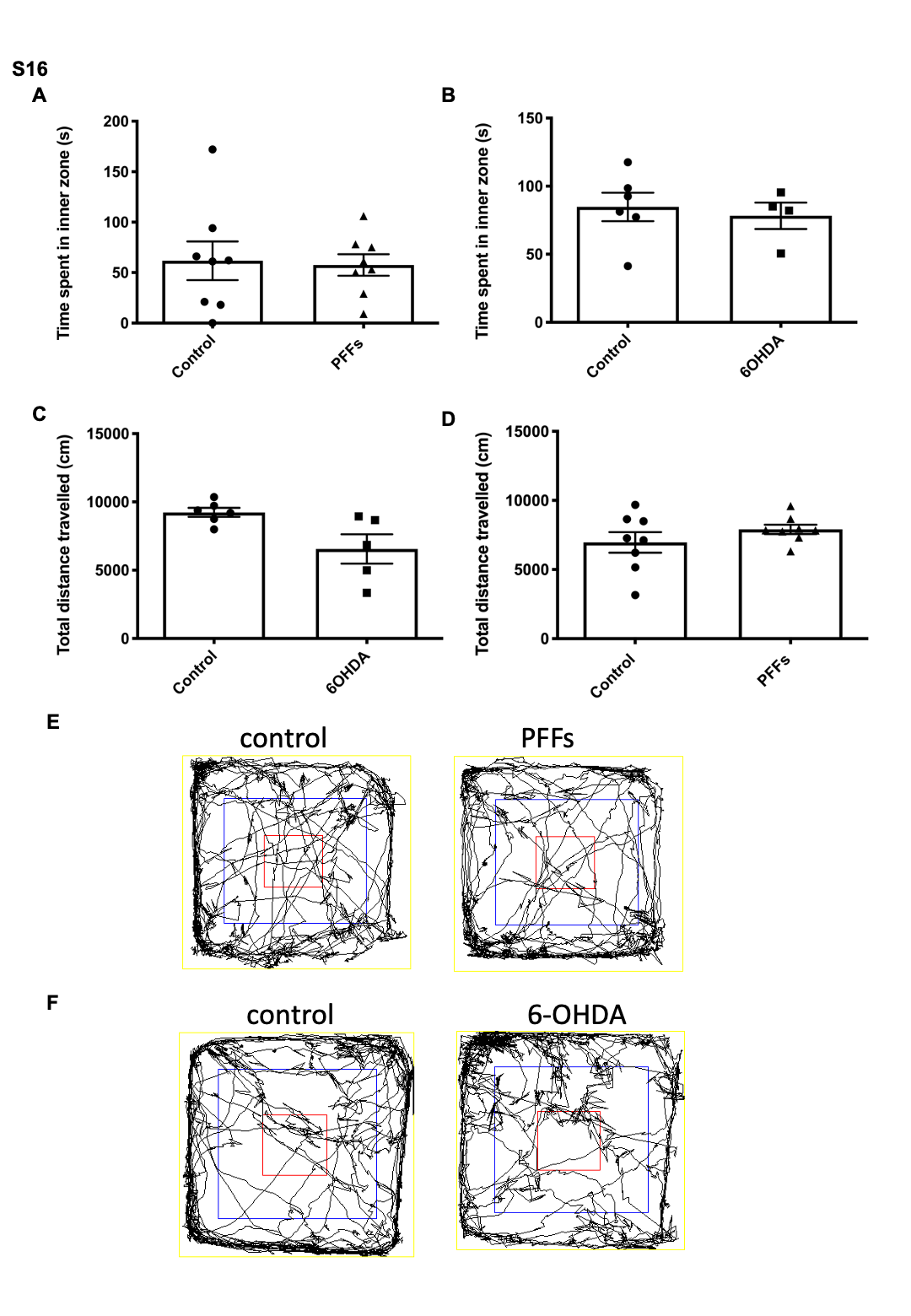

Supplement: Supplementary file 16 — Figure S16: A‐B) Measures of time spent in seconds inside the centre zone as an indication of anxiety‐like behaviour using the open field test shows no difference between control and 6‐OHDA groups or α‐synuclein PFF and control groups. C‐D) Land‐based locomotor activity was measured as total distance travelled in centimetres. Control and 6‐OHDA rats and α‐synuclein PFF and control rats travelled similar distances. Graphs show trajectories of representative E) Control and aSyn PFF, and F) Control and 6‐OHDA‐treated rats during the open field task. The yellow box represents the outer zone, the blue square is the middle zone and the red box indicates the inner zone of the open field arena. Data is mean ± SEM, statistical analysis used was two‐tailed unpaired t‐test (control n = 6, 6‐OHDA, n = 4​, control for PFFs n = 8, PFFs, n = 8). [file NAN-48-0-s011.tiff]
